# Polar Similars: Using Massive Mobile Dating Data to Predict Synchronization and Similarity in Dating Preferences

**DOI:** 10.3389/fpsyg.2019.02010

**Published:** 2019-09-06

**Authors:** Jon Levy, Devin Markell, Moran Cerf

**Affiliations:** ^1^Kellogg School of Management, Northwestern University, Evanston, IL, United States; ^2^Hinge Inc., New York, NY, United States; ^3^Media Lab, MIT, Cambridge, MA, United States

**Keywords:** online dating applications, decision making, homophily, big data, matching

## Abstract

Leveraging a massive dataset of over 421 million potential matches between single users on a leading mobile dating application, we were able to identify numerous characteristics of effective matching. Effective matching is defined as the exchange of contact information with the likely intent to meet in person. The characteristics of effective match include alignment of psychological traits (i.e., extroversion), physical traits (i.e., height), personal choices (i.e., desiring the same relationship type), and shared experiences. For nearly all characteristics, the more similar the individuals were, the higher the likelihood was of them finding each other desirable and opting to meet in person. The only exception was introversion, where introverts rarely had an effective match with other introverts. When investigating the preliminary stages of the choice process we looked at the consistency between the choice of men/women, the time it took users to make these binary choices, and the tendency of yes/no decisions. We used a biologically inspired choice model to estimate the decision process and could predict the selection and response time with nearly 60% accuracy. Given that people make their initial selection in no more than 11 s, and ultimately prefer a partner who shares numerous attributes with them, we suggest that users are less selective in their early preferences and gradually, during their conversation, converge onto clusters that share a high degree of similarity in characteristics.

## Introduction

Online dating has become one of the most popular methods for single individuals to meet and develop relationships (Madden and Lenhart, 2006; Valkenburg and Peter, 2007; Finkel et al., 2012). As early as 2005, over a third of single Internet users were using online dating services. Within the 2 years that followed, more new romantic relationships had begun as a byproduct of online services than through any other means, with the exception of meeting through friends (Finkel et al., 2012). The usage of mobile applications (apps) for dating has nearly tripled, and apps are predicted to continue growing in the following years (Juniper Research, 2015). Currently, dating apps exist for users as young as those in their teens and as senior as those in their eighties and nineties.

Traditional online dating sites (OkCupid, Match.com, JDate, etc.) focus on allowing users to create extensive profiles with photos and a multitude of fields for self-description. Typically, once a user creates their profile, they can search through the profiles of potential romantic partners in the hope of communicating and eventually meeting in person. Contemporary mobile dating apps (Tinder, Hinge, Bumble, etc.) use recommendation algorithms to present users with a stack of potential matches that are believed to have the highest likelihood of connecting in a meaningful way. On these apps, each potential romantic interest is displayed one at a time with a photo and basic information, such as age and location. A user can click on the profile being presented and see additional information. This may include height, religious beliefs, hometown, various interests, and a short bio. Users have the option to either reject or accept the person as a potential match but cannot view the next potential match until they have made a selection. Once two users confirm their interest in one another they are both notified and are able to communicate. By 2016, over 60% of the mobile app dating market included this type of selection process (Statista.com, 2016).

Many mobile apps are populated with information by pulling data from the user’s social media account (typically, Facebook), rather than having users manually fill out extensive profiles. This provides a wealth of knowledge previously unavailable for traditional online dating services. Additionally, this provides a higher confidence in the user’s identity, age, hometown, current city, occupation, education, etc. (Duguay, 2017). In the case of Hinge, which we will focus on throughout this paper, users are *required* to log in using Facebook, but can choose to manually enter additional information that is not available on Facebook, such as the type of relationship they are open to (i.e., “Casual”) or the religion they identify with. While many mobile dating apps do not require users to enter additional information about their height, political preferences, personality, etc., popular apps such as Tinder and Bumble have fields titled “about me” or “bio” that users commonly use to add these attributes. None of the popular mobile dating apps, including Hinge, require these data. However, Hinge does have dedicated fields for these attributes which make queries about them easy to evaluate.

Given that these apps make the preliminary selection of a partner a binary decision, they provide an environment that lends itself to quantitative decision making models. This is contrary to traditional online dating sites where the choice of a partner is harder to analyze and model due to the breadth and depth of information users are exposed to in the context of their decision making.

In this work, we investigate the selection process and look at the level of similarity between two individuals, across various attributes, as a driver of the ultimate match—that is, how attributes that pertain to a person (their height, religious affiliation, education, preferences, socio-economic status, or personality traits) indicate the likelihood that they will prefer to interact with others who share similar attributes. We show that people who are similar to one another tend to prefer each other and are more likely to actually engage in a conversation that leads to meeting in person.

We break the matching process into two stages and analyze each one separately in different sections of the paper. In the first half of the paper, we look at the choice to exchange contact information with another user after both people have expressed initial interest in one another, and some communication has happened through the app’s chat platform. This choice relates to the decision to potentially interact with the other person outside of the dating app.

In the second half of the paper, we investigate the binary choice to pursue an initial interaction with a potential candidate by merely signaling an interest in communication. In the world of mobile app dating this is typically noted as “swiping right” [on a picture of the candidate]. This choice happens first, typically followed by a conversation using the app chat platform, and then ending with a decision to interact outside the app sandbox.

Whereas the decision to swipe right is a binary yes/no decision reflecting a general interest in the other person, the exchange of information could be based on more knowledge about that person (including knowledge of expressed interest and potentially some additional information that was disclosed during the communication). Additionally, the choice to exchange contact information typically involves more commitment (i.e., disclosing personal revealing details). The choice to exchange contact information that leads to a meeting can also be seen as a choice between a broader set of options. The person is not just choosing whether they are interested in learning more about another individual online, they are choosing whether they want to spend time with them, at the expense of spending time with others, for what is typically a longer period. Therefore, this choice is seen as more involved.

Prior works looking at partner choices in the context of similarity and homophily—the tendency of individuals to associate and bond with similar others—have shown that such homophily permeates in marriage, friendship, and various interpersonal relationships (McPherson et al., 2001). Generally, the preference toward similar others was shown in the context of the similarity/attraction theory. The theory suggests that individuals tend to be attracted to those who are similar to themselves. This was demonstrated in the context of shared attitudes (e.g., views regarding family), personality traits (i.e., extroversion, neuroticism, etc.) (Youyou et al., 2017), physical attractiveness (Bruch and Newman, 2018), socio-economic status, religious beliefs, habits, ethnicity, and intelligence (Byrne, 1971; McPherson et al., 2001). Focusing on marriage, Schwartz (2013) suggested that partners tend to ultimately resemble one another on various features such as age, education, race, and more (Bruch and Newman, 2018). Contrarily, some research has focused on the notion that “opposites attract.” Observation by Winch and Goodman (1968) on compatibility among married couples suggested that some complementary, yet opposite, characteristics may lead to more successful long-term relationships. Recent research suggests that differences in personality can increase novelty and personal growth in the early stages of a relationship, leading to a more fulfilling dating experience (Finkel, 2017).

Additional works in the context of partner choice have explored the notion of an ideal standards model (ISM). ISM suggests that people consider a partner for a close relationship based on three factors: warmth-trustworthiness, vitality-attractiveness, and status-resources (Fletcher et al., 1999; Fletcher and Simpson, 2000), regardless of whether they possess those themselves. ISM predicts that people would end up more satisfied in relationships where their partner is perceived as aligned with their own ideal standard (Fletcher et al., 1999; Campbell et al., 2001; Buyukcan-Tetik et al., 2017) rather than if the partner is similar to them.

Some biological studies of mate selection seem to support the fact that the compatibility between partners is not likely to be at the level of exhibited attributes such as socio-demographic or socio-economic features, but rather at a genetic level. For example, Andersson and Simmons (2006) discuss immune system diversity as a driver of pairing. Another example suggests that opposite genetic profiles may drive attraction more than their manifested phenotypes (Santos et al., 2005). According to these studies, people with opposing characteristics may find each other attractive and desirable despite mounting personality differences because of attributes that are not directly visible to them.

While all these dimensions of a person could play a part in the pairing choice, due to the fact that dating is shifting from in person meeting to online discovery, the initial selection is now often based on basic information that is acquired remotely, in a short time window of seconds. This simplification reduces the number of dimensions a person can consider in partner selection and provides an opportunity to quantify the effects of specific attributes on the likelihood that couples will match.

In this work, we focus our investigation on features of a user’s mobile dating app profile and ask which are most effective in drawing a match between two people. Using the limited information provided to users when making a selection (name, a picture, location, school, relationship intentions, common friends) we try to estimate the likelihood of a pair choosing to exchange contact information and engage in a conversation outside the digital world. While we recognize that many encounters in the real world would still end up as a non-effective match, our scope is solely confined to the measure of initial success as defined by the app users—to translate the online correspondence into correspondence outside the virtual world (Gibbs et al., 2006).

Our work contributes to the growing body of literature identifying key characteristics in mating that lead to more desired relationships, offers tools to optimize the algorithms enabling the dating app world, and potentially aids in navigating the journey toward a successful match. Additionally, our results shed light on the app-based dating horizon which seems to reflect the preferred method of meeting potential partners for the younger generation (ages 16–38). This is the age group that yields the highest revenues in digital domains in the Western world (Smith, 2015) and, accordingly, is sought after by many corporations.

## Materials and Methods

### Data

Data were gathered from Hinge (Hinge Inc.; New York, NY, United States), a popular dating application used across the two most popular mobile platforms (iPhone and Android). Among all dating platforms (including non-mobile ones), Hinge is ranked 14, with 1.1% of total dating platform users. This included records for more than half a million users and hundreds of millions of entries prior to November 2015. Overall, the data reflect interactions among users in 38 cities in the United States, England, India, Australia, and Canada (see Appendix 1 for full list of cities). Our analyses focused on users within the United States as they make for the bulk of the data. We included only data from heterosexual relationships (i.e., a user who self-identified as male, who expressed interest in females) which reflect the majority of Hinge users.

### Sample Description

A user profile on Hinge has data that is pulled from social media (Facebook), entered by the user, inferred from the device used, or generated as a byproduct of the interaction within the app. Data fields include name, gender, age, education, height, and various other basic biographical information. Users are not required to complete all possible fields. Data such as height, education, and religious beliefs may be left blank. Ethnicity was selected by the user upon subscribing to Hinge. We only used ethnicity data in the context of name/initials similarity analysis, per Hinge’s request. Fields such as age, name, gender, education, number of social media connections and device type are populated automatically.

To estimate preference and likelihood of effective matching we excluded any users who, for a particular query, did not provide the specific data (e.g., only those who provided their religion were included in queries related to religion). We did not impute missing data given that this would require accessing individual user information rather than aggregated data, which we did not have access to. Additionally, we suspect that in the context of online dating, missing data may be indicative of a deliberate choice not to include the information (i.e., a short man deciding not to disclose his height, thinking this may increase his dating prospects) and therefore should not be altered. More so, when a user is selected based on missing information this in itself may be indicative of a preference. Imputing the data and drawing conclusions based on this may alter the determinations. Accordingly, our sample fluctuated in size depending on the queries used. Nonetheless, we were always working with hundreds of millions of entries.

In prior studies (i.e., Hitsch et al., 2005) populations of online dating participants were compared to general population statistics provided from surveys. These prior studies suggested that the percentage of men is disproportionately represented in the data. In our dataset, albeit slightly skewed toward men, the numbers were virtually the same. A possible match would not be affected by the number of options presented as users are faced with a limited set of users to choose from within a given day. This daily limit is determined dynamically from a combination of available pool size and the user’s level of activity. The pool ranges between 10 and 21 per day. Hinge’s value proposition at the time of the analyses emphasized trying to initially connect people who had Facebook friends in common but was not limited to this group.

Some concerns exist with regards to the accuracy of user data (Brym and Lenton, 2001; Madden and Lenhart, 2006) as users may misrepresent some attributes. In fact, based on a study by Toma et al. (2008) users indeed misrepresent their height, but not to a significant degree (about half an inch for men). To test for this bias we compared average male and female user heights to national averages in the United States (Fryar et al., 2012). Both male and female users on Hinge were, on average, slightly taller than the national averages (males: 71.1′ compared to national average of 69.3′, *p* < 10^–3^, *t*-test, Cohen’s D: 0.88; females: 65.3 inches compared to national average of 63.8 inches, *p* < 10^–3^, *t*-test, Cohen’s D: 0.77). This difference can be partially explained by exceptionally short users who may not declare their height and, therefore, are not represented in our queries. These differences, in conjunction with some users’ intentional misrepresentation of their height, would sway the averages slightly. However, since people match based on the provided information, regardless of whether it is true, we treated the height values as if they were accurate. With regard to age and gender, since data is pulled from Facebook, a user must be willing to go through the arduous process of changing their date of birth or gender on Facebook (including waiting for the information to update on the Facebook platform and then repopulating Hinge) in order for those to be represented differently. Although it is possible to do so, it seems unlikely that this would be a common occurrence.

### Queries and Analysis Tools

Data were pulled from the Hinge Database using a series of SQL queries, into large Tab-Separated-Value files. All user data were anonymized prior to the scientific inquiry. The academic members of the team had no access to the users’ personal information. All data usage was done in alignment with the Hinge license agreement^1^. Ethical review and approval were not required for the study on human participants in accordance with the local legislation and institutional requirements. No personal user communication was read or used in the study, and the only content that was observed—the exchange of contact information—was extracted using a regular expression that indicated whether such information was exchanged (yes/no). No text, user names, or pictures were available to the research team throughout the analyses.

### Typical App Record Entry

To open an account on Hinge, users begin by downloading the mobile app to their smartphone. Once they open the app users are prompted to create an account using their Facebook credentials. The user provides Hinge with access to basic information and images. These include name, gender, age, location, occupation, education, etc. (see Table 1 for a full list of fields).

**TABLE 1:** Fields used by the Hinge application for user analysis.

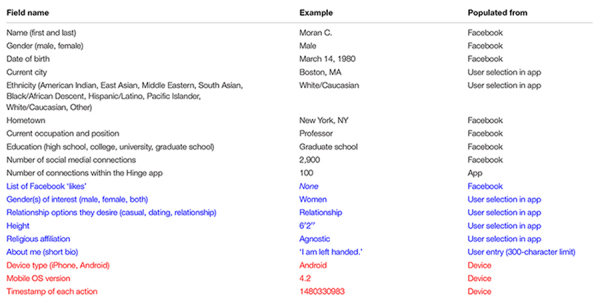

*Example of fields for an individual user. Data is populated from the information user enters into the Hinge application and from their Facebook profile. All fields in black are mandatory. Entries appearing in blue are optional fields that may or may not be completed. Entries in red are populated by the device per user and action (i.e., the rating of an individual by the user). A table is maintained per user that includes the list of all individuals rated by a user and the selection for that individual.*

Out of all users included in this study, 10% had all fields filled out, 61% did not have height filled out, 82% did not have “open to” filled out (“open to” indicates the type of relationship the user is interested in), 9% did not have education filled out, and 18% did not have a hometown filled out. To complete the registration processes users’ photos are pulled from their Facebook account. These photos can be reordered, replaced, or removed.

Once the initial account setup is complete, users are presented with a daily stack of potential matches for review. As part of standard operations, Hinge tracks additional data on the choices and the devices used to make the selections (in red in Table 1).

### Typical User Experience

A typical experience with Hinge involves a user opening the app using their smartphone and seeing their stack of potential matches. A match is reflected in the form of an image that a user is asked to swipe with their fingers. Swiping an image to the left indicates “no-interest,” whereas swiping to the right indicates a desire to communicate with the person further. A match is defined by both users, independently and asynchronously, selecting the respective partner as someone they seek further communication with. If a user matches with another user, they unlock a chat feature of the app. Once the stack of potential matches is complete, the user can wait until the stack is refilled (up to a day) while chatting with users who already matched with them.

Not all information about a potential match is readily available at first glance. Access to religious beliefs, relationship type interest, height, hometown, and a brief bio (comprised of up to 300 characters; 106.4 ± 81.1; mean ± SD) require a user to scroll down.

Not all matches lead to conversations. Even fewer lead to an exchange of a telephone number or other means of communication outside the app.

For the purposes of this study we tracked three characteristics relative to each variable we tested:

#### Potential Match

The count of every potential couple that *could* occur. Included in this group are both: (1) matches where one user saw another user and rejected them (note that in this case Hinge will not display the choice to the second user since a match cannot occur unless both users respond positively), and (2) matches where one user selected the other, and the second one had the opportunity to select/reject the first user (regardless of whether they selected them).

#### Conversations

Given that not all matches result in conversation, we define a conversation as an interaction where each person messages the other twice (i.e., Alice messages Bob once. Bob then responds. Alice messages again, and then Bob responds again). This is termed a “four way” by the online dating industry. For the purposes of this paper, any communication less than that is deemed “not a conversation”.^2^

#### Effective Match

Since we cannot track a user’s behavior once they leave the app, we consider the exchange of means of communication outside the app as the metric of success (i.e., a phone number exchange). This suggests that both users are interested enough in one another to continue talking and potentially meeting in person. This also suggests that a level of comfort and perceived safety has been achieved to advance the communication to the level of more identifiable information. From this we define the Effective Match Rate (EMR) as the percentage of effective matches relative to the total number of potential matches.

### Social Ratio Metric

To compare psychological attributes, we used standard popular metrics of personality (Little, 2014). One such measure is introversion. As we cannot judge directly if a person is introverted or extroverted, we used a user’s Facebook friend count as a proxy. This is based on the fact that introverted individuals are likely to have fewer friends relative to the average number of friends in their peer group (Amichai Hamburger and Vinitzky, 2010). When examining an average user’s friend count, we found that the numbers vary based on the city a person lives in, their gender, and their age. For example, Hinge users who are older women have far fewer Facebook friends than younger women (on average, a 45-year-old woman has 360 fewer friends than a 25-year-old woman). For this reason, we evaluated each user in comparison to others of the same age, gender, and city.

A user’s “Social ratio” is defined as the ratio between the number of Facebook friends they have and the average number of Facebook friends that users of the same age, gender, and city have. Users who have a Social Ratio lower than 1 veer toward introversion while those who have a Social Ratio above 1 veer toward extroversion.

### Education-Related Data

To evaluate the effects of school ranking, liberal arts education, and NCAA conference participation, we matched the Hinge/Facebook school entries to those of the Integrated Postsecondary Education Data System (IPEDS) database. These were then used to map users’ schools to National Center for Education Statistics, which allowed for binning based on academic similarity (test scores, school’s focus disciplines, etc.).

Not all schools listed in the Hinge database were found in the IPEDS database. Some schools are not accredited and would not be included in IPEDS. In other cases, users misspelled their school names or simply made up fake schools (e.g., “The School of Hardknocks”). Schools outside of the ones recognized were treated as missing data. Eighty one percent of users in the sample attended the same 1,500 schools.

For categorization of colleges by institution type (Liberal Arts College or National University) we looked at the rankings (U. S. News and World Report, 2016) of the top 200 schools by institution type.

For categorization of colleges by Division 1 NCAA sports conferences, we compared the IPEDS database to online listings of school participation (see Appendix 2 for breakdown). As Ivy League is an NCAA conference designation, we also used these data to define Ivy League schools.

## Results

To examine the ways Hinge users pair into a relationship we looked at data from 421,690,471 potential matches. These reflect data from over one million users, with an equal distribution of men/women. Before users can chat and exchange contact information, they need to review one another’s profiles. To estimate whether users were swiping based on the readily available information in a profile (i.e., the image) or whether they were viewing additional information in an extended profile, we looked at the average time spent scanning the candidate. Men engaged with the profile for 6.7 ± 4.7 s. Female users spent significantly more time making their choices (11.1 ± 6.9 s; *p* < 10^–3^, *t*-test. Cohen’s D: 0.82).

The time spent viewing profiles suggests that most of the selection occurs based on immediately available cues such as aesthetics, visual presentation, and basic notable information that is readily accessible (i.e., occupation or mutual friends). Given that the estimated average time it takes to saccade to an item on an average screen size is about 200 ms (Mackay et al., 2012), we estimate that a typical user had up to 33 pieces of information that they were able to capture before making a decision. Face recognition assessment, emotion assessment, and attraction preferences require 1–5 s to process (Todorov and Uleman, 2002; Willis and Todorov, 2006; Todorov et al., 2009; Venkatraman et al., 2015) leaving the male user anywhere from 1.7 to 5.7 s to incorporate most of the textual information into a decision. Female users would have 5.1–10.1 s to incorporate the additional information. This is sufficient to typically read 2–4 fields while also scrolling through the profile page (Dyson and Haselgrove, 2001).

Given that faces are likely to draw the users’ attention first, followed by the text (Milosavljevic and Cerf, 2008; Cerf et al., 2009), we suspect that visual information was processed for a longer time during the decision. In order to generate our own baseline assessment and not rely solely on prior works, we also ran a query on user rating data to determine the typical scan time for a profile. Response times were measured as seconds elapsed between the previous rating and the current rating time. These baseline estimations included 1,000 randomly selected users (500 males and 500 females). The average scan time in our data was 700 ms longer than the literature standard, with an average of 7 s allotted to a profile. This is enough time to load and review all the personal information on the user’s front page. Although male users spend less time per profile, they engage with more profiles, leading to similar amounts of total time spent reviewing potential matches as female users do.

The likelihood that any two potential matches would have a conversation is 0.51%. This is the total number of “four way” conversations (2,148,947) divided by all potential matches across the entire database. The average EMR across the entire dataset is 0.12% (508,989 effective matches divided by all potential matches). This means that for every 4.23 people that a user chats with, they will exchange contact information with one. In comparison, work that created dummy Tinder profiles and measured how many of them were selected, shows that roughly 0.6% of males who select a female get selected by her (or 1 out of every 167), and that 10.5% of females who select a male get selected by him (or about 1 out of every 95) (Tyson et al., 2016). This means that, on average, men say “yes” to 17.5 times as many women than women do men.

It is worth noting that given the size of our dataset, even small effects are significant. That is, even a change of fractions of a percent in EMR is likely to be significant and meaningful. We therefore used Cohen’s D as an additional metric to quantify the effect size when necessary. We used a cutoff of 0.8 to note large effects and 0.2 to note small effects throughout.

Below we characterize a number of individual attributes and their effect on the matching likelihood:

### Education

While one might think that the choice of partner in a rapid binary selection processes is skewed toward more superficial properties—typically aesthetics—we tested the correlation between cognitive and more long-term aspects of the match and their effect on the outcome. One such aspect is education. A typical higher education in the United States lasts 4 years. The selection of school reflects a choice of location, socio-economic status, intellectual goals/abilities, and also, at times, shared values (e.g., a choice to go to a small liberal arts college trades size of student body for type of education. Alternatively, attending a competitive technology-based institute of higher education may have a notable difference in ratio between men and women).

These preferences, tradeoffs, and considerations reflect a person’s perspective and values and can thereby also influence their preferences in dating. To gain insight into these characteristics, we evaluated the influence of university type on effective matching. Specifically, we examined the designations of schools (Liberal Arts and Ivy League) and the school’s participation in an NCAA conference on EMR.

### The Influence of Attending a Liberal Arts College (LAC) on Effective Matching

Liberal arts colleges attempt to impart students with a well-rounded education in the arts and sciences (Grove, 2015). They focus on developing intellectual capacities and broad knowledge. These colleges tend to be smaller. For the purposes of this research we compared schools ranked by the U.S. News and World Report (USNWR) in the LAC category with top-ranked National Universities and with colleges not present in the rankings.

When both men and women attended a liberal arts college their EMR was 0.20%, a 38.0% increase compared to cases where only one attended an LAC and the other attended a ranked non-LAC. Similarly, attendees of LACs were 34.6% more likely (0.20% EMR versus 0.15%) to match with each other than with people from unranked schools. Among users who both attended a non-LAC ranked by USNWR, the EMR was 0.17%. This is higher than people from unranked schools matching with each other and people from ranked non-LAC matching with people from unranked schools, both having an EMR of 0.16% (see Table 2).

**TABLE 2:** Liberal arts college.

**College**	**Total possible matches**	**Conversations**	**Effective matches**	**Conversation probability**	**EMR**
Both went to LAC	329,003	2,652	674	0.81%	0.20%

LAC vs. non-LAC ranked	3,588,852	22,174	5,326	0.62%	0.15%

**Both LAC vs. different ranked**				**30.5%**	**38.0%**

LAC vs. non-LAC unranked	8,893,225	56,275	13,536	0.63%	0.15%

**Both LAC vs. different ranked**				**27.4%**	**34.6%**

Both non-LAC ranked	12,872,132	86,337	21,553	0.67%	0.17%

**Both non-LAC ranked vs. different ranked**				**8.6%**	**12.8%**

Both Unranked	80,304,037	535,216	130,100	0.67%	0.16%

Unranked vs. ranked	69,598,483	452,860	112,092	0.65%	0.16%

**Both unranked vs. ranked**				**2.4%**	**0.6%**

**Overall**	166,692,507	1,099,239	269,745	**0.66%**	**0.16%**

### The Influence of Attending an Ivy League College on Effective Matching

In the United States, the prestige of attending an Ivy League college is paramount to many other academic markers of success, as it has implications on social status, future career, and potential earnings (Rivera, 2011). With so much value placed on attending these institutes, we asked whether attendees of these schools select one another and match more effectively with one another.

When both users attended an Ivy League school, they had an EMR of 0.27%. This is more than double the average EMR of 0.12% and is 64.3% more frequent than if only one person attended an Ivy League school and the other person attended any other institute of higher education (0.27 vs. 0.17%; see Table 3).

**TABLE 3:** Ivy League colleges.

**Ivy league**	**Total possible matches**	**Conversations**	**Effective matches**	**Conversation probability %**	**EMR %**
Both Ivy league	105,390	911	289	0.86	0.27
Ivy vs. non-Ivy	6,223,089	38,728	10,388	0.62	0.17
Same vs. different				38.9	64.3

### NCAA Sports Conferences Affiliation as It Relates to Effective Matching

As users did not directly state if they had sporting allegiances, we reviewed the NCAA conference their college participated in as a proxy for such preference. In situations where two users attended schools that participate in the same NCAA conference, there was a positive increase in probability of effective matching versus situations where the users had dissimilar conferences. The increase ranged from as little as 7% for those students from “Big Ten Conference” schools to as much as 91% for students from the “West Coast Conference.” On average, the probability of effective matching increased by 21.1% if both users shared such affiliation (see Table 4).

**TABLE 4:** NCAA sports conferences.

**NCAA conference**	**Increase in probability of effective match %**	**Significance (*p-value*)**
Northeast Conference	152	0.003
Metro Atlantic Athletic Conference	147	10^–4^
Ohio Valley Conference	121	0.004
West Coast Conference	91	0.001
Conference USA	84	0.007
Southeastern Conference	74	10^–4^
Mid-American Conference	70	0.003
Ivy League	64	10^–4^
Atlantic 10 Conference	59	10^–4^
Southland Conference	58	0.099
American Athletic Conference	58	10^–4^
Missouri Valley Conference	50	n.s.
Patriot League	37	0.004
Big West Conference	34	0.036
Sun Belt Conference	33	n.s.
Mountain West Conference	28	n.s.
Big 12 Conference	25	0.003
Atlantic Coast Conference	20	0.006
Colonial Athletic Association	17	0.046
Pac-12 Conference	15	10^–4^
Big Ten Conference	7	0.028
Big East Conference	2	n.s
Southern Conference	−2	n.s

Given their small sample size, we excluded from the list conferences with attendance below 50,000 people (see Appendix 3 for list of school excluded). We note that similarity in sports allegiance may simply mean that the two users are, in fact, in the same school, but an overwhelming percentage of Hinge users are already graduates of college suggesting that, unless they stayed in the same city where their university was, they are likely being presented with a wider variety of people.

### Mobile Device Type as It Applies to Effective Match Rate

It has become a common phenomenon for consumers to align themselves with brands that they love and use (Allison and Uhl, 1964; Kressmann et al., 2006; Tuškej et al., 2013). These brand allegiances can have subtle impacts on the way people behave and the choices they make (Barnett and Cerf, 2015). These brands are also shown to reflect and correlate with personality types and characteristics (Grant, 2017). Our dataset included the mobile operating system each user was using (iPhone or Android). We tested whether there is a relationship between dating preferences and operating system selection (see Table 5).

**TABLE 5:** Mobile device type.

**Device**	**Total possible matches**	**Conversations**	**Effective matches**	**Conversation probability %**	**EMR %**
iOS/iOS	298,215,755	1,574,075	370,053	0.53	0.12
Mixed: 1 Android and 1 iOS	98,290,340	461,698	112,187	0.47	0.11
Android/Android	8,280,795	41,583	10,788	0.50	0.13
Users have same phone	306,496,550	1,615,658	380,841	0.53	0.12
iOS/iOS vs. mixed				12	8.72
Android/Android vs. Mixed				7	14.14
**Same vs. different**				**12.2%**	**8.9%**

The data suggests that users who have the same smartphone (both iPhone or both Android) experience an increase of 8.9% in effective matches versus users with dissimilar phones, although the knowledge about the operating system used by the other user is not overtly accessible. Android users had an EMR of 0.13% (a 14.14% increase over mixed), followed by iPhone users who had an EMR of 0.12%. Users who had dissimilar phones had an EMR of 0.11%. The differences between all device types were significant (*p* < 10^–3^, *t*-test).

### User Initials as They Correlate With Effective Matching

One popular scientific claim known as “implicit egotism” suggests that similarity to oneself generates appeal/attraction in the context of names that resemble one another (Pelham et al., 2002; Jones et al., 2004). One finding from this line of research suggests that people who have the same initials (i.e., Mark Goffman and Maya Goffer) are 8.8% more likely to marry one another than those with differing initials (Jones et al., 2004). Whereas the original research was conducted on a dataset of 14,534 people, we now have data from over 421 million potential matches, so we tested the results in a more robust way. User names on Hinge appear as the complete first name and the first initial of the last name (i.e., Albert E.). Users with the same initials had, on average, an 11.3% increase in effective matching compared to those with dissimilar initials (0.13% versus 0.12%; *p* < 10^–3^, *t*-test; Table 6). While implicit egotism has been controversial in the literature and the effect size is small, our dataset allows for a testing and verification of the hypothesis. Our results hold upon controlling for religious affiliation, which could have been a driver of disproportionate name selection (i.e., some religion have preference for some names that may increase their proportion in the dataset).

**TABLE 6:** User initials.

**Condition**	**Compatibility count**	**Conversations**	**Effective matches**	**Conversation probability**	**Effective match probability**
Identical Initials	1,736,588	9,575	2,332	0.55%	0.13%
Differing Initials	419,807,709	2,138,336	506,439	0.51%	0.12%

### Desired Relationship Type as It Relates to Effective Matching

Common assumption pertaining to users of dating apps who select potential partners based on little preliminary information is that they are likely to pursue casual romantic relationships. Although we have no data on the nature of the relationship once the users exchanged phone numbers, many users will disclose the type of relationships they desire within the app. Users can select none, one, two or all of the following three options: “Casual,” “Dating,” or “Relationship.” Users whose relationship intentions are aligned have an increased rate of effective matching (Table 7). When both users state they desire a “Relationship” (understood as a committed relationship) their EMR is 0.20% compared to only 0.13% when only one user states a desire for a relationship. Similarly, when both state an interest in “Dating” the EMR is 0.19% compared to 0.14% when only one person expresses an interest in dating. Those users who are both looking to be “Casual” have an effective matching rate of 0.15%, which is lower than when both are looking for a “Relationship” and both are looking for “Dating” but still higher than the 0.13% EMR when only one person states an interest in being “Casual.” All differences are significant (*p* < 10^–3^, *t*-test). Note that there are overlaps within the mixed options (i.e., “Dating” and “something else” could end up being the same as “Casual” and “something else,” if in this example the “something else” ends up being “Dating” or “Casual”). Therefore, comparison between the mixed options were not complete.

**TABLE 7:** Desired relationship type.

**Conditions**	**Total possible matches**	**Conversations**	**Effective matches**	**Conversation probability %**	**EMR %**
Both interested in “Causal” relationship	1,487,847	10,565	2,277	0.71	0.15
Male Casual, Female not	38,410,837	223,604	50,628	0.58	0.13
Female Casual, Male not	11,613,164	62,372	13,565	0.54	0.12
Casual vs. non-Casual	50,024,001	285,976	64,193	0.57	0.13
Both interested in “Dating”	17,194,118	122,238	32,763	0.71	0.19
Male Dating, Female not	61,745,450	336,695	81,824	0.55	0.13
Female Dating, Male not	65,375,580	373,637	91,728	0.57	0.14
Dating vs. non-Dating	127,121,030	710,332	173,552	0.56	0.14
Both interested in “Relationship”	17,078,132	124,039	34,251	0.73	0.20
Male Relationship, Female not	58,210,924	312,425	76,751	0.54	0.13
Female Relationship, Male not	67,451,437	392,506	97,711	0.58	0.14
Relationship vs. non-Relationship	125,662,361	704,931	174,462	0.56	0.14

In both the cases of “Dating” and “Relationship” women more often match with men who have dissimilar interests (0.14% EMR) than men who match with women of dissimilar interests (0.13% EMR; *p* < 10^–3^, *t*-test). In the case of the choice of “Casual” the opposite is happening: men who are looking to be “Casual” and women who are not have an EMR of 0.13% versus women who want to be “Casual” and men who do not (0.12% EMR; *p* < 10^–3^, *t*-test).

### Religious Belief in Comparison to Effective Matching

Religious belief has been a long-standing point of contention for couples getting together (Blackwell and Lichter, 2004; Mahoney, 2005; Hitsch et al., 2010). Conversely, common religious affiliation can increase the chances of shared values and interests. In an era where we see a growing departure from faith, one may ask how important is it for couples to share the same religion?

People who either do not list their religion or have no religious affiliation make for a large pool of potential matches. However, looking at the data from users who state their religious affiliations, we see that users who share the same religion have an average 97.5% increase in their EMR (to 0.21%) compared to people with mixed religions (0.11%; Table 8). Chances of effective matching for two people of the same religion is as high as 0.94% for Muslims (856.5% chance over Muslims and non-Muslim) or as low as 0.17% for Catholics (50.0% chance over Catholics and non-Catholics). The smaller the community representation in the data, the more likely they were to effectively match with people of the same religion. Hindus make for only 327,911 potential matches in our dataset and have 0.61% EMR. Similarly, Muslims make for only 3,741 potential matches with 0.94% EMR. In contrast, Christians have 8,558,535 potential matches and 0.20% EMR and Jews have 8,026,793 potential matches with 0.30% EMR. Notably, these numbers are not proportional to the numbers in the larger population but are aligned with census data of younger app users, primarily in urban environments. Our dataset does span a wide range of cities and locales and, accordingly, reflects a representative offering of religions and political views.

**TABLE 8:** Religious beliefs.

**Religion**	**Total possible matches**	**Conversations**	**Effective matches**	**Conversation probability %**	**EMR %**
Both Catholic	7,000,149	49,750	11,960	0.71	0.17

Male Catholic, Female not	37,098,926	183,370	41,955	0.49	0.11

Female Catholic, Male not	43,902,466	218,159	50,334	0.50	0.11

Catholic vs. non-Catholic	81,001,392	401,529	92,289	0.50	0.11

Both Christian	8,558,535	69,302	17,231	0.81	0.20

Male Christian, Female not	32,553,015	160,043	37,282	0.49	0.11

Female Christian, Male not	47,126,801	232,754	54,875	0.49	0.12

Christian vs. non-Christian	79,679,816	392,797	92,157	0.49	0.12

Both Hindu	327,911	7,172	1,988	2.19	0.61

Male Hindu, Female not	4,234,491	16,401	4,471	0.39	0.11

Female Hindu, Male not	3,968,157	20,874	5,413	0.53	0.14

Hindu vs. non-Hindu	8,202,648	37,275	9,884	0.45	0.12

Both Jewish	8,026,793	83,604	24,237	1.04	0.30

Male Jewish, Female not	31,322,221	136,600	34,314	0.44	0.11

Female Jewish, Male not	37,413,003	151,240	34,261	0.40	0.09

Jewish vs. non-Jewish	68,735,224	287,840	68,575	0.42	0.10

Both Muslim	3,741	138	35	3.69	0.94

Male Muslim, Female not	890,443	3,511	805	0.39	0.09

Female Muslim, Male not	738,226	3,437	788	0.47	0.11

Muslim vs. non-Muslim	1,628,669	6,948	1,593	0.43	0.10

Both Spiritual	1,501,120	11,747	3,817	0.78	0.25

Male Spiritual, Female not	15,831,114	81,991	22,144	0.52	0.14

Female Spiritual, Male not	19,681,091	116,478	32,240	0.59	0.16

Spiritual vs. non-Spiritual	35,512,205	198,469	54,384	0.56	0.15

Both Agnostic	1,630,120	10,169	2,977	0.62	0.18

Male Agonistic, Female not	25,140,010	117,015	30,036	0.47	0.12

Female Agonistic, Male not	16,474,358	83,530	21,388	0.51	0.13

Agnostic vs. non-Agnostic	41,614,368	200,545	51,424	0.48	0.12

Both Atheist	567,176	3,937	999	0.69	0.18

Male Atheist, Female not	16,927,835	76,374	17,883	0.45	0.11

Female Atheist, Male not	7,934,231	37,146	8,889	0.47	0.11

Atheist vs. non-Atheist	24,862,066	113,520	26,772	0.46	0.11

Both Other	368,060	2,084	566	0.57	0.15

Male Other, Female not	13,950,519	70,846	17,183	0.51	0.12

Female Other, Male not	7,728,977	38,670	10,011	0.50	0.13

Other vs. non-Other	21,679,496	109,516	27,194	0.51	0.13

**Total same choice**	**21,427,114**	**185,449**	**48,645**	**0.87%**	**0.23%**

**Total difference choice**	**400,175,176**	**1,962,256**	**459,933**	**0.49%**	**0.11%**

For all religious affiliations, except for Judaism, women of a particular religion had an EMR of 0.13% with men outside their religion. Non-Jewish women were 5.7% more likely to match effectively with men outside their religion than their male counterparts. Jewish women and non-Jewish men had a low EMR of 0.09% (significantly different than the 0.30% Jewish women-men pairing; *p* < 10^–3^, *t*-test).

### Introverts and Extroverts

Western cultures tend to emphasize outgoing or extroverted personalities (Allik and McCrae, 2004; McCrae and Terracciano, 2005). The general tendency that is often aligned with extroversion suggests that extroverts gain energy from engaging with others, whereas introverts prefer more intimate social interactions (Amichai Hamburger and Vinitzky, 2010). We investigated whether users match most effectively with others who share their level of introversion/extroversion.

**FIGURE 1:**
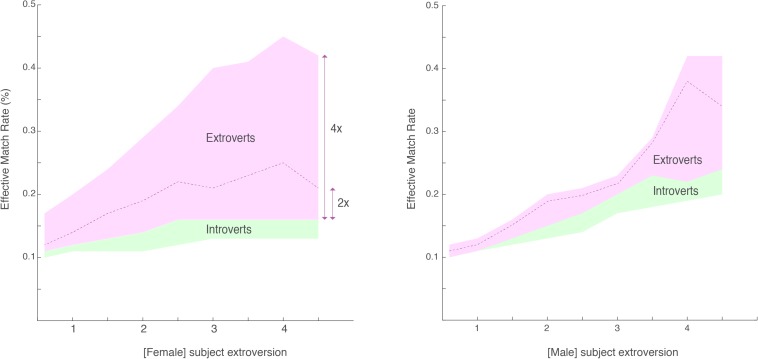
Effective Match Rate as it relates to female **(left)** and male **(right)** social ratio. Female/Male extroversion level (measured in number of times above the average number of Facebook friends for their city, age, and gender) and their likelihood of finding a match. Men whose social ratios are four times more than the average, for example, are likely to have a higher EMR with women whose social ratio is three times above the average. The top right points and bottom left points are identical since they reflect the extreme matching of both genders.

In our dataset, introverts rarely match effectively with other introverts, but when at least one member of a potential match is an extrovert the EMR rises significantly (Figure 1). Men who have a social ratio of 2 and above (that is, they have twice as many friends as the average) are significantly more likely to effectively match with women of every level of the extroversion-introversion spectrum. This effect increases with men’s social ratio.

Effective match rates increase with social ratios for both genders. These effects are stronger when women have a social ratio above 2. These women had an EMR 53.8% higher than women with a social ratio below 2 (*p* < 10^–3^, sign-test). These effects are amplified disproportionately when men’s social ratios are also above 2. For example, men’s EMR increased by 71.9% when their social ratio was above two compared to below 2. When men’s social ratio was four times higher than the average their EMR increased by 157.5%.

### Height

**FIGURE 2:**
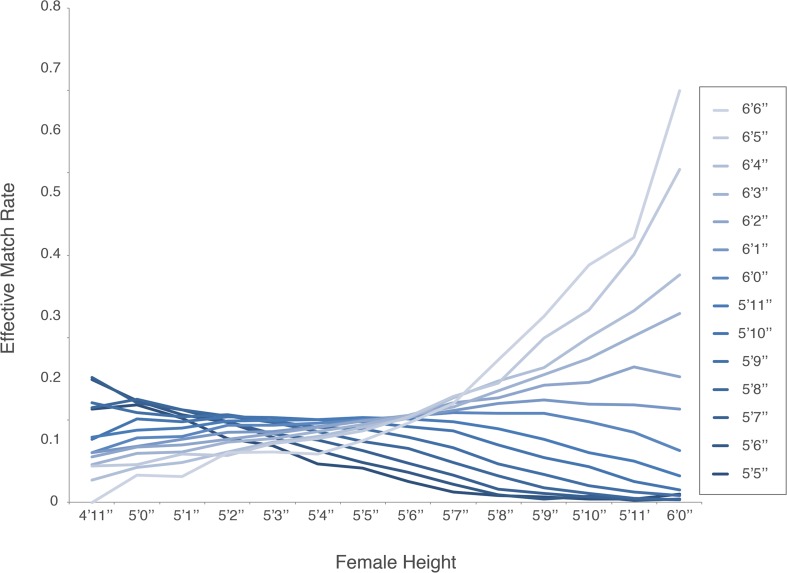
Effective match rate as it relates to a female height (from 4′11′′ to 6′0′′) and male height (5′5′′ to 6′6′′).

Height has been shown to have an impact on multiple facets of dating choices. The literature suggests that taller men have a higher chance of generating initial interest among women, that heterosexual couples where men are several inches taller than women are happier, and that shorter men are likely to marry later in life (Weitzman and Conley, 2014; Sohn, 2015). Our analysis uncovered a more complex relationship between men’s and women’s heights. When examining which height combinations have the highest EMR we found that optimal selections are dependent on a combination of gender and height (Figure 2).

Effective Match Rate (function of male height *m* and female height *f*) is:

$$E\,M\,R\,\left(f,m\right)=\sum_{i=0}^{2}\sum_{j=0}^{2}a_{i\,j}\,f^{i}\,m^{j}$$

where $a_{ij}=\left(\begin{array}{ccc} 3,172 & -90.01 & 0.64\\ -99.49 & 2.83 & -0.02\\ 0.78 & -0.02 & 0 \end{array}\right)$

Notice that the coefficient matrix is nearly symmetric with a minor weighted influence toward female heights.

Accordingly, the optimum female height for a male user (function of male height *m*):

$$E\,M\,R^{\prime }\,\left(m\right)=-\frac{1}{2}\cdot \frac{\sum_{i=0}^{2}a_{1\,i}\,m^{i}}{\sum_{i=0}^{2}a_{2\,i}\,m^{i}}$$

And the optimum male height for a female user (function of female height *f*):

$$E\,M\,R^{\prime }\,\left(f\right)=-\frac{1}{2}\cdot \frac{\sum_{i=0}^{2}a_{i\,1}\,f^{i}}{\sum_{i=0}^{2}a_{i\,2}\,f^{i}}$$

The optimum women heights for matching with men of any height are in the range 5′1′′–5′6′′ (66.7% of the female population in their 20 s).

## Decision Making Process

While men and women must both select each other in order for an effective match to occur, their strategies of selecting a partner may differ. Our results show that individuals who share common attributes (religion, education, etc.) are likely to match effectively at the end of the courtship journey. To address the decision making process in full, we further investigated the initial stage of the matching journey.

Presumably, users can identify partners who share traits with them early on or start with a wider net of options and converge to traits similar to theirs. That is, users can either be very selective in the initial choice or accept many possible matches and hope that among the numerous options there are also partners who are similar to them.

Strategies of selection can be attributed to a specific preference or to lack of certainty about the choice and the hope that additional information will increase the information. Similarly, strategies of rejection can be attributed to lack of interest, the elimination of highly appealing options due to a feeling that the person is “out of one’s league,” or as a preventive measure to avoid future rejection when they do not match.

Given that at the end of the match process people effectively matched with others who largely shared traits with them, we investigated whether men and women also exhibit similar strategies in the early stage of the matching processes. That is, are the similarities in outcomes the consequence of similarity in early choice strategy, or a gradual convergence?

To investigate the early binary choice, we tried to fit the decision using classical prediction model. Whereas most decision making models (i.e., the Drift Diffusion Model) typically estimate the “response time” and the “accuracy” (Fehr and Rangel, 2011) of a decision, we replaced the “accuracy” with “consistency” (in the absence of “ground truth” for individuals, we measured how likely a user is to agree with the selection of prior control group users). We fitted men/women’s choices and looked at their similarities in time, consistency, and other attributes that can be inferred from Drift Diffusion Models (DDM).

To test similarities in decision making we asked the following questions: (1) Do users tend to be similar in their preferences early in the choice process? That is, do men/women first choose the same people or do idiosyncrasies arise in preliminary selection? (2) Do men/women spend similar time on the early choices, or are there differences in the early stages that potentially shed light on the alternative trajectories in their thought processes? (3) Do users exhibit “streaks” of consistently saying “yes” (or “no”)? This would suggest a less focused search method, and that the ultimate similarity in effective matches are adopted while the individuals converse or after they have learned that there was an initial mutual desire to interact.

To estimate consistency, we assigned a likelihood to be selected (0–10) to 100,000 randomly selected users (5,000 men), who were seen by at least 200 people. We calculated the likelihood by looking at the chance of a person being selected by people who previously viewed them. Simply, if a user was seen by 100 individuals and was selected 20 times, we would score the person’s desirability as 2.

**FIGURE 3:**
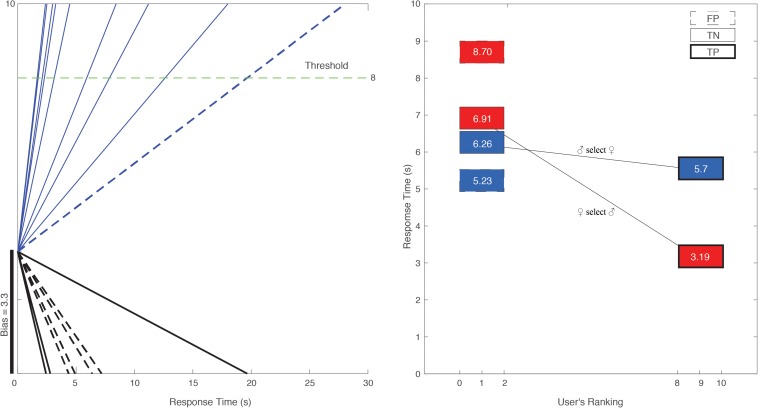
**(Left)** A male user’s partner selection modeled through DDM. We show (*x*-axis) the time the user took to make a selection when faced with candidate profiles. The user selected multiple women whose rank was above 8 (blue solid lines) and rejected multiple women whose rank was below 8 (black solid lines). We mark the selection as “True Positive” (“hit”) and the rejection of lower ranked women as “True Negative.” “Miss” (“False Negative”) would be a rejection of a woman ranked above 8 (dashed black lines) and “False Positive” would be the selection of a low-ranked woman (dashed blue line). Based on the user’s prior choices we can also estimate his “bias.” The user tends to accept 33% of the women he sees. His response time is shorter when selecting a high-ranking woman and rejecting a low-ranking one. **(Right)** Mean response time for men selecting women (blue) and women selecting men (red) who are ranked 8–10 (True Positive; solid bold lined square) or rejecting ones ranked below 2 (“True Negative”; non-bold solid lined squares). Accepting a user that others typically reject (“False Positive”) is depicted with dashed lines.

When testing for the consistency of a user’s selections, we evaluated how much a choice aligns with those of prior viewers. When viewing a candidate whose score was high (i.e., 9), the expectation is that a new viewer would select them as well. Similarly, a user whose score is 1 was likely to be rejected. When a new user rejects a 9 or selects a 1, they are inconsistent with prior viewers and reflect a unique view. We analyzed only those extreme cases: users whose score was above 8 or below 2 (see Figure 3). Users whose scores were closer to the middle (i.e., 5) suggest an idiosyncratic evaluation by viewers. Excluding these users biases our estimates toward higher consistency, in alignment with prior works showing that individuals are generally more likely to be consistent in ranking content presented visually (Cerf et al., 2007). This consistency assessment allowed us to learn whether men/women are similar in the way they make their decision.

Figure 3 depicts the trial duration in 17 trials for subject 2 (male) and threshold of 8 (indicating that any individual they see whose score is above 8 is expected by our model to be selected). The subject indeed selected 8 of the choices. He rejected four of the women he was presented with, whose ranks ranged between 1.6 and 1.9 (below the threshold) and accepted one woman who was ranked below the threshold. The response time for the below-threshold acceptance was notably longer (28.03 s). The acceptance of all high-ranking women was much faster, with the highest-ranking woman (ranking 8.9) selected after 4.91 s and the fastest rejection (lowest ranking woman 1.6) after 2.42 s. The subject’s personal bias was rather low compared to other men (3.3 on a 0–10 scale, suggesting that a woman whose rank is below 3.3 would normally be rejected by the person). The DDM assumes a random walk and not a linear trend toward the goal, however given that we only know the trajectory based on the final outcome we plot those as straight lines.

### Estimating Consistency

Men spend 5.70 ± 0.3 s accepting highly desired women, whereas women spend nearly half the time (3.19 ± 0.8 s) accepting a man who prior women ranked highly (Figure 3; *p* < 10^–3^, *t*-test; Cohen’s D: 3.94). While women are faster in selecting the desired men, they are slower in rejecting the undesired ones. Women would spend 6.91 s before rejecting a man that other women ranked 2 or below. Men assessing profiles of undesired women spend 6.26 s on this rejection. In alignment with the DDM we can term the acceptance of a desired person “True Positive” (“hit”) and the rejection of an undesired one “True Negative.”

False Positive is an acceptance of a person who is ranked below 2. Women take longer to do so (8.7 s) than men (5.2 s). Simply put, women are faster in accepting an attractive man, while men are faster in rejecting an unattractive woman. Altogether, men seem to spend equal time on all profile assessments, whereas women are notably different in assessing desired men from undesired ones.

Subjects occasionally spent an unreasonably long time deciding (e.g., 295 s before a swipe). This could be due to the fact that they looked away from their phone or used the application in a non-typical way. To improve our estimates, we tried removing trials with lengths above the mean + 1 SD. These trials constitute 8.1% of the total. With these trials excluded, all choices decreased in similar proportions and, altogether, show average differences of 1.3 s in all attributes. For example, the average response time for women decreased to 2.1 s when selecting a desired man (drop of 1.09 s). The response time decreased by 1.1 s for the selection of desired women by men. The only notable deviation from the prior results was the rejection of undesired men by women, which decreased to 6.1 and now seems more within the realm of other choices rather than an outlier.

### Model Fit

Using our definition of True/False Positives/Negatives we could now fit our data with a DDM to estimate the time to decide and see if men/women seem to employ similar strategies in their initial selection. DDM typically assumes that a choice is binary and has two possible outcomes: select the person (1) or reject the person (0). The normalized range of 0–10 often assumes that the initial state of the selection is at 5, but this is not always the case. One way to assess the initial state is by estimating an individual’s likelihood of selecting *an* option regardless of the one faced (e.g., a user that says “yes” to 90% of choices would start at 9 whereas one that says yes to only 40% of the choices would start at 4). We term this initial setting the *bias*. We assessed the *bias* for each individual prior to fitting the model based on at least 50 prior choices they made. Next, we estimated the time a user spent making each choice. Our estimate essentially aligned with the standard DDM equation:

(1) d⁢x=τ⁢d⁢t+ε

where τ is the evidence or information the user has in order to make their choice between the two options at any time point *dt* (their Threshold for a yes/no), and ε is a noise term. The noise, on average, should integrate to zero.

To estimate the decision making process we looked at a single choice made by each of our 100,000 selected users. Each row corresponds to one selection (i.e., a male user viewing a female user and rejecting her). This yields a table of 100,000 rows with each row having the following four fields: the user’s gender (male/female), the rating of the user they were viewing (0–2 or 8–10), the choice they made (accept/reject the user), and the response time for the choice rounded to the nearest integer.

We note that adding a ranking of the selecting user in addition to that of the selected user would have allowed us to add an additional feature to the decision model. However, this would deviate from typical DDM. Therefore, we did not incorporate this information (a study that did focus on the interaction between the attractiveness of the selecting user and the selected user was done by Bruch and Newman (2018).

**FIGURE 4:**
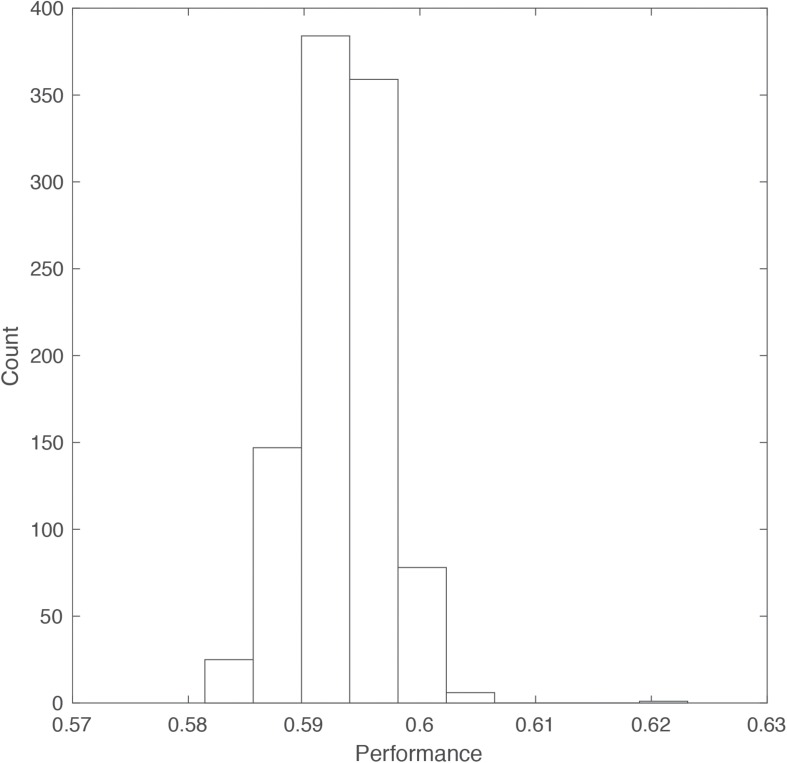
Histogram of DDM performance accuracy. For 1,000 repeated selections of subsets of the data we used DDM to estimate the time a user would take to make a consistent decision (rejecting a user who was previously ranked 0–2 or accepting one who was ranked 8–10).

We randomly selected 70% of the data as a training set for a classifier (Linear Discriminant Analysis classifier, using Matlab’s *fitcdiscr* function) while holding out the remaining data for testing. Then, we estimated the response time for each choice of the remaining data based on the gender, target user’s rating, and selection. Simply, we tried to predict the time it would take a user to accept/reject another user based on their ranking. This aligns with decision making models that suggest a tradeoff between speed and accuracy (rejecting an unattractive user or accepting an attractive one). We compared our response time predictions to the correct ones and averaged the resubstitution error (ranging from 0 to 1) to get an estimate of our classification accuracy. We repeated this entire process 1,000 times to generate a distribution of accuracies (Figure 4).

Our model average prediction accuracy was 59.3%. That is, knowing a user’s gender, we can predict the time they would take to accept/reject another user with nearly 60% accuracy, without additional prior knowledge. Adding the *bias* term to the equation increases the accuracy to 61.05%.

Men are 3.09 times more likely to select a desired woman when they respond faster than the average (20.36% compared to women at 6.58%). When looking only at the extreme cases (e.g., acceptance of a person who is ranked below 1) men accept a low-ranking woman (False Positive) 4.18% of the times and women do so 9.42% of the times. However, whereas a man will spend 8.3 s on the selection, women increase their time to 14.20 s. These are significantly higher (*p* = 0.01 for all comparisons, *t*-test) than all other choices.

### Streaks

Finally, we moved from looking at the choice as a single outcome to looking at the choice sequence (“streak”) in order to see if there are differences in the strategies that men/women employ when looking at multiple choices. This, in our model, would fit in the bias term as it includes *memory* of prior information in each choice. We focused on the tendency to go on “Mate Binging” when a user essentially accepts/rejects multiple options in a sequence. This typically suggests less attention to each individual choice. It is important to note that the design of the app at the time of the study limited a user to 21 potential matches a day (the exact limit was determined by an algorithm, with an average of 15 matches per day). If a user had a streak, or “binge,” of 45 “yes” choices in a row, it would have been completed over at least 3 days.

When examining selection streaks (the number of potential matches that users respond to consecutively with the same response—either all yes or all no) we see an interesting difference in strategy between men and women.

**FIGURE 5:**
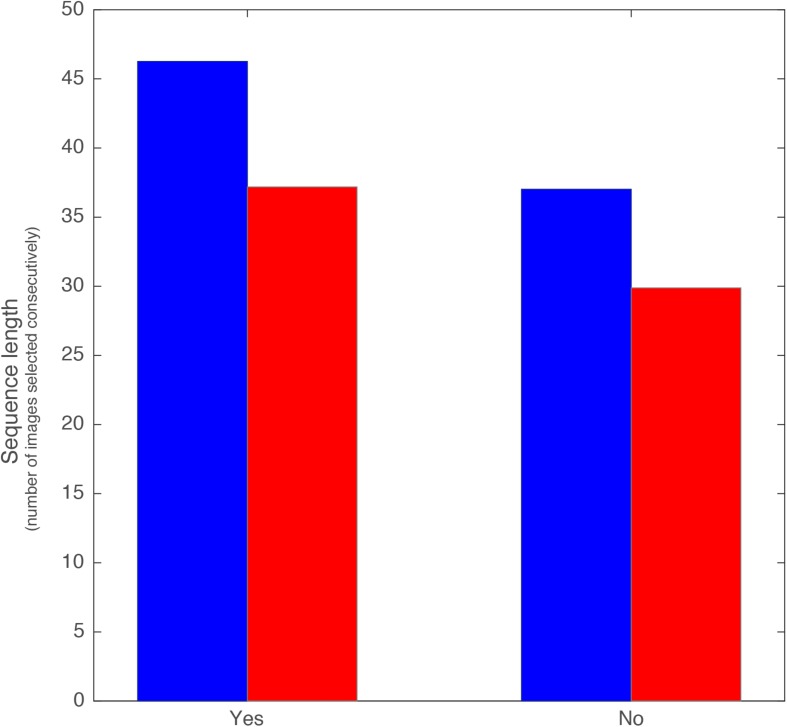
Length of continuous sequence of accepting **(left bars)** or rejecting **(right bars)** a person by women (blue) and men (red). All *t*-test between all conditions show significance below 10^–13^.

The average longest “yes” streak for women is 46.26 (Figure 5). However, few women are likely to go on such a streak of saying yes (1.3%), whereas the majority of women (43.1%) are likely to have their longest streak of saying “no.” The average longest “no” streak is 37.02. Men are divided between those whose longest streak is saying “yes” and those whose longest streak is saying “no” nearly equally (26% “yes” and 24% “no”). Men say “no,” on average, to 29.9 women consecutively.

### Initial Choice Strategy

Taken together, our results suggest differences in strategies between men and women in the initial stages of the choice process across all metrics evaluated. Therefore, one can assume that the narrowing of the choice happens later in the process, after the initial screening has happened. Given the selection strategies we observed, it is likely that users start the choice process by focusing on salient attributes such as visual features and basic observable characteristics (i.e., characteristics that suggest similarity in taste). We implicitly rely on the convergence of women being more specific in their acceptance and men being more specific in their rejection.

Later on, the couple presumably focuses on the similarities and highlights the more nuanced features that would ultimately yield emphasis of religion, school, sports teams, and so on. This may be done either through the conversation, or as the users spend more time investigating the profile of the people they matched with.

## Discussion

In this work, we assessed the likelihood of two individuals communicating and potentially dating each other using mobile online applications. We estimated the potential of a pair matching based on various attributes such as education, religion, or psychological traits.

Our results show that individuals tend to gravitate, online, toward partners who share similar traits to them.

This tendency to select an effective match with partners who share traits, is shown in the realm of education, relationship preference, religious preferences, height, and essentially all attributes we investigated. Prior research has shown that people choose friends who are similar to them in a wide array of characteristics: age, race, religion, education level, socio-economic status, political leaning, aesthetic rating, or even handgrip strength (Dunbar, 2018). This is true for hunter-gatherer groups as well as modern societies. Our data therefore support the prior works.

In the context of mobile online dating, this similarity in traits is particularly interesting given that it is true even if those traits are covert on the mobile app (some of the parameters by which the pair end up being similar are not available to them at the time of the choice). This suggests that users end up figuring out who would be similar to them either by using silent and hidden visual cues or through the conversation following the initial choice.

Some parameters that users match on are likely to be the outcome of the geography or lifestyle settings. For example, iPhone users tend to have higher than average income than Android users. Therefore, the fact that we see an increase in EMR across iPhone users could simply reflect that users from similar socio-economic levels gravitate toward one another. More complex examples could be the indication of whether an individual is, for example, an introvert. This information is not overtly exposed to anyone at a brief glance of an image, yet influences the matching likelihood and, accordingly, must be communicated in alternative ways. Recent works in computer vision and psychology suggest that some personality attributes can in fact be gathered merely from the visual imagery (Cerf et al., 2008; Wang and Kosinski, 2018).

The understanding that similarity is predictive of effective matching is useful as it allows for an improvement of the matching process and the scaling of the success rate of dating. The application of this can be either in the implementation and optimization of the matching algorithms to offer users more similar candidates to choose from, or it can allow users to make their selection in a more efficient way, as they would know their likely preferred match.

Given that (1) online dating is currently a major setting by which individuals meet, and (2) studies on couples who meet online suggest that online dating yields higher rates of satisfaction from the relationships and lower rates of breakups than traditional matching (Cacioppo et al., 2013), any improvement in the ability to identify a preferred partner is likely to lead to an increase in satisfaction. Borrowing from the literature on decision making and psychology, we can assume that if fit between products and personality increases happiness (Matz et al., 2016), then a fit between two individuals stands to yield an even greater increase in overall satisfaction. This is assuming that a choice of a partner is more personal and long-lasting, and that human connection trumps connection to non-human entities (companies, objects, etc.; see Mentovich et al., 2016). Indeed, prior works looking at similarity between people have shown that such alignment between individuals sharing psychological traits could, in fact, reflect an underlying neural synchrony that is likely to yield matching behaviors across various domains such as purchases (Barnett and Cerf, 2017) or political opinions (Barnett and Cerf, 2018).

### Comparing Our Results to Existing Data on Matching

We compared our results to those of two domains that investigated the choice of a partner: assortative mating and traditional dating (i.e., meeting offline, speed dating, web-based online dating, etc.).

Assortative mating suggests that, in biology, partner selection is guided by tendencies to identify individuals with similar attributes (Jiang et al., 2013). While assortative mating typically focuses on genetic selection, the mating literature has shown prior evidence that seeking similarity or homophily in partners is often reflected in the phenotype level as well. Assortative mating holds across almost every characteristic that can be assessed in our data.

Looking at the early selection process alongside the ultimate match outcome, we see that users are not only similar to each other in their features, but also employ similar decision making strategies. Accordingly, we are able to use data on preferences by individuals to model the choice of a test group and predict some of the choice parameters.

Comparing our results to the literature on traditional dating, we are able to provide a unique reflection on the existing works. A notable advantage of our work is the size of the dataset investigated. Access to a dataset of this scale by academics is rare and nearly impossible without collaboration with industry. We used this opportunity to compare our results to existing data on web-based online dating, speed dating, in person dating, survey data, and matchmaking. Given that, as we noted earlier, in the last couple of years it is presumed that most dates in the Western world involve an online component—primarily online apps—it is useful to see how our results compare to earlier works. Table 9 summarizes the literature compared.

**TABLE 9:** Literature.

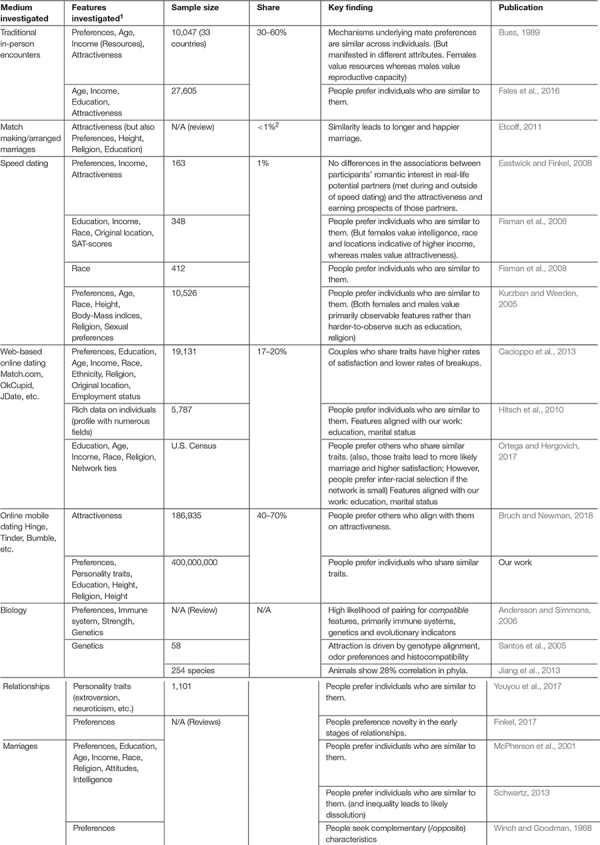

*Alignment of our work with existing literature looking at similarity between partners in mate and dating selections on various features. The list samples works that show similar method or dataset to ours. ^1^List includes: Preferences, Personality traits, Education, Age, Income, Race, Ethnicity, attitudes, Intelligence, Attractiveness, Height, Body-Mass indices, Religion, Original location, SAT-scores, Network ties, Sexual preferences, Immune system, Strength, Genetics. ^2^In the Western world, but higher numbers in religious and traditional communities, (primarily outside of the U.S.). If we include a friend ‘introduction’ it climbs to 5–15%.*

Hitsch et al. (2010) also measured the effective match rate and estimated the parameters that led to a likely match. In their work the data came from online dating that is not on mobile apps. The data for an individual was richer since users were asked to generate a profile where they filled in additional details, such as body type (lean, muscular, over-weight, etc.), marital status (single, divorced, widowed, etc.), and numerous additional biographic information. However, the number of users and interactions is significantly smaller compared to ours. When comparing only the attributes that our datasets share, we note that the results seem to match. Hitsch’s work has also shown that both men and women want to meet a partner with similar attributes. For example, users prefer similar education levels (the results were estimated using a maximum likelihood of the fixed effects using binary logit model, with the assumption that the first-contact and rejection costs are zero). However, Hitsch’s comparison was between years of schooling rather than type of education. In Hitsch’s data, women have an overall strong preference for an educated partner but also have a relatively small tendency to avoid men who are more educated than them. Men generally shy away from educated women altogether. Comparing our data to that of 10,526 participants in a dating service known as HurryDate (Kurzban and Weeden, 2005), which involves actual meetings alongside survey data, we see that participants have a preference for partners from the same age and religion categories. The focus in the HurryDate study was on age, body-mass indices, race, and marital status—all of which we do not use in our dataset. On height, which both our datasets compare, we see that both ours and Kuzban’s work show a preference for men to be taller than women. Altogether, Kurban’s work, which also shows a preference for couples who are similar in attributes to match, aligned with our results.

The works of Fisman et al. (2006, 2008) looked at another coupling method—speed dating—and showed that individuals tend to prefer others who come from regions comparable in population size. The other parameters measured involved income, race, SAT scores, and other sets of information which we did not have access to. This, too, aligns with our data, although our results are biased by the matching algorithm’s tendency to suggest local/nearby candidates, thereby altering the likelihood of choosing a partner from other locales with different population sizes.

Our work also aligns with existing literature on arranged marriages. Looking at the overall likelihood of marriages to last (measured by years until divorce), arranged marriages of couples who share religion, education, or who have height differences similar to the ones noted in our results, have a higher likelihood of lasting longer. These couples are also the preferred option by matchmakers (Etcoff, 2011). Note that arranged marriages are mostly popular outside of the United States, making the comparison to Hinge skewed (since virtually all the data we examined come from the United States).

Other prominent works in the field of match assessment focused on race (Fisman et al., 2008), income (Buss, 1989; Fales et al., 2016), weight, and prior declared preferences by the individuals (see Eastwick and Finkel, 2008 for a comprehensive discussion), all of which we had no access to in our dataset.

Note that race, specifically, was shown to be a significant driver of coupling in prior works, with the majority of pairs selecting a partner from the same race [however, the increased use of online dating has been shown to correlate with greater numbers of inter-racial couples (Ortega and Hergovich, 2017), presumably due to increase in encounters between people who would otherwise never met, creating connections with unassociated social groups and reducing the isolation of groups]. Prior work on dating apps has shown that White/Caucasian men and Asian women are the most popular selections (Bruch and Newman, 2018), while Black/African Descent women and Asian men receive fewer matches.

We did not analyze race similarity (appears in our dataset as “Ethnicity”) aside from examining the frequency of initials within a certain ethnicity. However, given that the strongest driver of coupling in our data was religion, which often correlates with ethnicity, we suspect that the similarity in ethnicity/race is likely to be true in our data as well.

When examining works on the ISM (Fletcher et al., 1999; Campbell et al., 2001) the user attributes we examined can be viewed as representing the ISM characteristics (warmth-trustworthiness, vitality-attractiveness, and status-resources) in a variety of ways. It would stand to reason that those who have the same education, for example, may also share status (especially in higher degrees). Similarly, two people of the same religion could be seen as aligned on their trustworthiness virtue. Similar height could be a marker of attractiveness, etc. Therefore, the selection of an individual could be seen as a selection aligned with ISM attributes.

Given the high agreement between all the datasets on the outcomes, Finkel (2017) suggests that if a person is interested in optimizing their chances of identifying an effective match, mobile online dating should be the preferred option. That is, given that the results from all works are similar, but the scale of online dating is bigger, using dating apps would yield the highest return on the time and effort invested. Importantly, research looking at the algorithms used by online dating platforms to offer improved matching outcomes has shown that these algorithms prove unsuccessful in predicting a likely effective match based on stated preferences (Finkel et al., 2012; Joel et al., 2017). However, given our results we can suggest that potentially including in those algorithms a weight-function that increases the likelihood of successful coupling by individuals who share certain attributes instead of focusing on stated preferences may prove beneficial in yielding a greater number of effective matches.

### Matching Attributes

The initial likelihood of a conversation occurring between two users in our dataset is 1 in 200 (0.51%). This is already substantially higher than the number of conversations a user is likely to spark on an average day (i.e., commuting in a metropolitan area, dining at a restaurant, or having a drink at a bar). To study the nuances of EMR beyond this baseline, we estimated the various features in our set and their independent contribution.

First, we assessed the likelihood of a couple choosing each other based on their preference toward liberal arts colleges. Users who are both from liberal arts colleges matched effectively almost 40% more than pairs where only one was from such a college. We reason that users who went to similar schools likely participated in similar activities or had similar interests, which could be reflected in their photos and biographical information on the app. Therefore, in future communication on the app they would likely have a shared starting point for connection and conversation and have a sense of familiarity which, in turn, could become a driver for future communication (Shalizi and Thomas, 2011). Less clear is why liberal arts students would match more with attendees of unranked schools than with non-liberal arts colleges. One theory could suggest that students of more competitive institutes of higher education would match more effectively with each other than with those that do not make the ranking. Another possible explanation is that men prefer women who are less educated than they are as suggested by Hitsch et al. (2010).

Looking further at education, we see that Ivy League students show similar effective match patterns to liberal arts college students. Matching among Ivy League students is nearly 65% higher when one of the users is not from these eight schools. Given that the eight Ivy League schools have, historically, been pitted and compared with each other for decades it would seem likely that people who have earned the prestige of attending them may look for similar partners.

The likelihood of NCAA conference participants effectively matching (ranging from 7% increase above average EMR for those students from “Big Ten Conference” schools to as much as 91% increase for students from the “West Coast Conference”) is high as well. This could be explained partially by the fact that schools that participate in the same NCAA conference are generally in the same geographic area, or that the topic of sports tends to act as an introductory topic of conversation. Generally, sports teams and players have long had a culture of devotees that connect and engage around them—from European football clubs to American sports bars. People select their social circles, weekend activities, or the colors of their outfits based on their affiliation to sports teams. This may also drive users to end up sharing similar preferences in our dataset.

Mobile devices and their relationship to effective matching have potentially interesting implications. User similarity in mobile preference yields a higher likelihood of an effective match; however, the effect is small (0.01% increase. Cohen’s D: 0.21). While our dataset has almost 300 million potential matches between iPhone users, the mean effective match rate was 0.12%, which is lower than that of Android users (0.13%). These numbers are higher than the EMR of users with different devices (0.11%). While some research suggests that a person’s mobile device reflects potential trends, character traits, photo-taking preferences, and writing style (Grant, 2017), an alternative hypothesis to the reason behind such match proportions could be merely geographical or socio-economic. Indeed, data from Twitter usage, which contains the device used for the post, suggest that iPhone users often cluster on the coasts and within major cities in the United States, whereas Android users are elsewhere (Edwards, 2014). A likely combination of all theories — usage of mobile device pertaining to a certain income, geography, and style — could be the driver of these matches. In itself, this result is curious given that it is assumed that the choice of device usage during the matching process is not relevant to a partner’s choice, nor is it revealed explicitly during the conversation.

Testing the phenomenon of implicit egotism on the national level by exploring the effects of users’ initials on effective matching is consistent with results from prior work (Jones et al., 2004) in showing that individuals with shared initials tend to gravitate toward each other. Our results show that effective matching among those individuals is 11.31% higher than among individuals who do not share the same initials. Compared to population results showing that marriage percentage across such individuals is 8.81% above chance, our results trend in the same direction.

It is important to note that skeptics of this phenomena have expressed concern that implicit egotism may be driven by name frequency in specific regions and ethnic groups since users who share a specific faith or ethnicity are more likely to share a common last name. Given that we *did* have ethnicity data for users, we investigated the effect of ethnicity in this context. In our data, users identifying as Jewish are 670% more likely to have a last name beginning with “S” and 223% more likely to have a first name beginning with “J” than a user identifying as Muslim. Similarly, users identifying as East Asian are 152% more likely to have a last name beginning with “L” than users self-identifying as White/Caucasian. Under such conditions the effect of implicit egotism may be a byproduct of a preference for religious and/or ethnic identification. As we did not have full access to ethnicity/race data in our analyses, we normalized our results by religious affiliation proportions as a proxy for ethnicity. The effect remains the same.

When examining religious orientation as it relates to effective matching, it is not surprising that people matched more often with users that had a shared religious affiliation (EMR = 0.21%) than with users whose religions differed. Religions that had smaller representation on the app had the highest rate of effective matching. Muslims, with only 3,741 potential matches (0.0009% of all possible matches) had an EMR of 0.94%. Hindus (0.08% of all possible matches) had an EMR of 0.61%. Both are significant (*p* < 0.01) in comparison to the average EMR of 0.12%. Every other group had over 500,000 potential matches but a lower EMR. Interestingly, the data show that, across religions, men were more selective (2.97%) than women in dating outside the religion. The only exception is Judaism. This may be accounted for by the fact that in Judaism the religion passes from the mother, not the father (Mishnah, Kiddushin, 200AD). As such, it may reflect a greater pressure on Jewish women to date men who share their religion.

When looking at the stated preferences in relationship type, it greatly reinforces the value proposition of mobile dating apps when people who have aligned desires actually have higher effective matching rates (EMR = 0.15–0.20% depending on desired relationship type). If a user desires a committed relationship it would reason that they are more likely to engage in conversations with those who have aligned preferences. Additionally, it may be the case that those who are interested in, for example, casual relationships would have different conversation styles and different sets of needs from the conversation. These disparate styles may be intuited from the conversation and drive the ultimate EMR.

Investigating the relationship between introversion/extroversion and matching shows that introverts rarely connect with other introverts. While the users initially show matching preferences for each other based on profile features, neither is likely to start a conversation. If one of the users is an extrovert, we see a significant increase in effective matching (71.9–157.5% increase in EMR). This effect correlates with the level of extroversion the two users exhibit (higher score in extroversion above the mean correlates with increase in EMR; *r* = 0.52, *p* = 0.03). Two extroverts are more likely to engage in a conversation leading to an effective match than if only one of them is extroverted. While social traits were studied extensively in the context of engagement in group relationships (Cain, 2013), their effects on dating preferences were not investigated thus far to the best of our knowledge. This work suggests a potential connection between personality and dating preferences that is different from those offered in the personality literature. This may imply that although introverts are able to “step out of character” in some social settings, the difficulty with dating is that they are interacting with strangers and have no basis of familiarity.

One should consider that this social ratio could actually function as a proxy for other measures of a user’s characteristics. For example, these may reflect how attractive a person is. Given that existing research shows that more beautiful people are more likely to find mates (Langlois et al., 2000), we explored this option as well. Our data do not support the theory that attractiveness is the driver for friend count. That is, if this alternate theory was correct, we would expect that people generally match with others with the same social ratios. This is not the case. Men with very high social ratios match with women who have very low ratios in high proportions and vice versa (Figure 1). Although social ratio is not a perfect proxy for introversion and extroversion, it provides a reasonable estimate and valuable insight into a link between personality traits, and effective matching.

Previous research on height as it relates to dating suggested that men and women prefer mates who have a specific relative height to their own (Pawlowski, 2003). Aligned with prior works, our data show a complex relationship between men’s and women’s heights (Figure 2). Women seem to prefer men taller than them but, given that height follows a normal distribution, there is not an unlimited supply of extremely tall men. As a result, we see a scarcity effect manifested in the data: women of a certain height may match most effectively with men of a specific height, but those same men may match effectively with women of a different height. Given that information in the matching experience is not fully available (i.e., a tall woman, for example, is not aware of the number of taller men and the likelihood of their emergence) the model for a user’s preference on height is skewed by the available resources. This drives the equation for matches among people of similar heights to be non-linear. Therefore, while we do not rule out the large contribution of height to desirability — especially for men — this does not guarantee an overall increase in EMR as suggested before.

### Early Decision Making Process

Beyond examining the EMR which reflects the outcome of a choice, conversation, and complex decision making heuristics, we looked at the attributes of the early decision process. These attributes reveal the considerations that go *into* the choice set and, presumably, affect the ultimate EMR. We focused on looking at whether men and women exhibit similar choice strategies with respect to consistency, response time, and streaks.

When comparing selection time (the time necessary to evaluate a potential partner), men’s and women’s strategies demonstrated significant variation in making an affirmative selection but were consistent when declining individuals. Women took a significantly longer time to accept a person that others typically rejected. That is, the tradeoff between speed and consistency is notably hindered with women on the side of accepting a low-ranking man, and notably improved when rejecting one. Men’s selection time is consistent for acceptance/rejection.

Given that Hinge caps the number of choices per day, making a streak of, for example, 45 “yes” choices to occur over multiple days, we could not estimate the choice memory/bias in full. While we think that a user’s 16th choice is influenced by their 15th choice, there is a chance that they are days apart in reality. With that in mind, we looked at streaks to see if they are similar among men/women. Women’s average longest “yes” streak is 46.3 choices long. Fewer women are likely to go on such a streak of saying “yes” (1.3%). The majority of women (43.1%) are likely to have their longest streak of saying “no.” The average longest “no” streak has 37.02 choices. Men are equally divided between those whose longest streak is of saying “yes” and those whose longest streak is saying “no” (26% “yes” and 24% “no”). Altogether they tend to alter their opinion more frequently and say “no” on average, to less than 30 women consecutively (Figure 4).

Importantly, we assume that the potential partner to choose from appears at random. However, given that the options come from a pool of candidates that are tailored by a matching algorithm, we cannot rule out the possibility that a “yes” streak is the outcome of a successful algorithm that rendered a sequence of ideal choices. Our intuition is that a long streak is likely a reflection of a user’s behavior. This is especially true when comparing genders, as the same matching algorithm is at play.

It is noteworthy that Hinge’s limit on the number of selections a user can exercise within a day is likely to strengthen the robustness of our results. That is, while the decision making strategy a user exercises in a finite domain could be different than the one made in an infinite choice horizon, we expect that having a limited number of attempts at a successful effective match would yield a more thorough vetting process. This is supported by recent data pertaining to the selection strategy employed in online web-based dating (Tyson et al., 2016; Bruch and Newman, 2018).

Additionally, while the limitation on the number of candidates a user sees each day may change the strategy they employ for the choice, it is unlikely to affect the chances of actually meeting the pool of users in a certain geolocation. That is because (1) Hinge extends the pool of candidates offered beyond merely the Facebook “friends of friends” when the pool of options is exhausted, and (2) including a user’s 2nd and 3rd degree connections within a certain geolocation is likely to incorporate the majority of users in that location. Put differently, if for example, a user lives in Toronto and is faced with a choice of another user on a dating app such as Tinder or Bumble, it is likely that the person they are viewing is also in their “friends of friends” circle on Facebook and therefore a potential match on Hinge as well. That is simply because of the estimates on the number of degrees of separation between any two individuals on Facebook. Facebook research shows that any two Facebook profiles are, on average, 3.5 degrees apart, and that this number likely decreases to 2–2.5 if the friendship circle is confined to a geolocation (Edunov et al., 2016).

Although our results reveal differences between the genders in selection style, these differences are minor when examining their overall outcomes. A striking result that emerges from our analyses is how consistent people are and how less unique their choices are compared to perception when it comes to partner selection. The fact that a simplified model based essentially on prior selections by users can predict both the choices and the response times of multiple individuals with accuracy of nearly 60% suggests that people are more predictable in their preliminary choices (accepting individuals that peers liked and rejecting ones that they did not) than often stated. Therefore, the idiosyncrasies and the convergence to similarities presumably happen later in the communication.

### Limitations

Our work has a few limitations. First, given that our dataset relies heavily on Facebook as the platform populating the user profile, it is important to note that existing works looking at the alignment between a user’s online and actual character are not perfect. While it is unlikely that a person would be entirely different on their online profile (as they are likely to be called out by their friends for such discrepancy) studies show that users do tend to exaggerate various attributes of themselves on their public image. Our study is, therefore, bound by the variance between the actual user profile and the depicted one. These differences are likely to be particularly pronounced in the context of extroversion (Amichai Hamburger et al., 2002; Mitchell et al., 2011).

Second, some attributes in our dataset have notably fewer samples than others and therefore should be interpreted accordingly. However, with the exception of NCAA and religion (within which only Muslims had low count) all our metrics included at least 100,000 potential matches and typically included over 1,000,000 samples if not one order of magnitude more.

A third limitation could be attributed to the way we define an effective match. We concluded that a match is effective when the two people in the conversations exchanged contact information. However, there could be alternative ways of setting a meeting that do not involve these. In order to maintain our decision to not read user messages and merely use regular expressions to infer if contact information was exchanged, we decided to refrain from including those alternative modes of setting an offline encounter (i.e., using the mobile app exclusively for all communication). Our results, therefore, act as a lower boundary to the proportions of effective matches that happened in our dataset and could be adjusted if future work could observe the conversation’s content.

Fourth, a large number of user profiles did not include all possible fields. Therefore, our results may be skewed toward individuals who were willing to disclose certain details. Additionally, the results are reflective of a complex selection process where missing information may or may not play a significant part. We do not know whether the inclusion of more information on a specific user would have increased/decreased the chances of them effectively matching and are limited to estimates within a specific attribute rather than across attributes.

Finally, despite the alignment of our results with various other types of dating sources (i.e., speed dating, actual meeting, survey data, match-making, and arranged marriages), it is noteworthy that our analyses are based solely on data collected from the Hinge mobile app, and caution should be exercised when generalizing the results to other mobile dating apps and populations. Some notable differences between Hinge and other prominent dating platforms pertain to the user demographics and choice architecture. For example, Hinge’s demographics is primarily heterosexual and therefore may not generalize to homosexual communities. Similarly, Hinge’s matching protocol does not impose rules on which gender is required to initiate a conversation or impose a time limit to a communication. Those differences may alter the choice dynamics. Some may strengthen our results (i.e., Hinge’s limit on the number of choices per day may make each choice more deliberate) and some may weaken our results (i.e., the requirement for additional fields in the profile may drive some users away from using the platform). We illustrate the key differences between Hinge and other leading mobile dating apps in Table 10.

**TABLE 10:** Popular mobile data apps.

**Characteristic**	**Hinge**	**Tinder**	**Grindr**	**Bumble**
Estimated percent of US online dating market engaged with app at the time of the study	1.1%	25.6%	6.3%	3.6%

Popular location	Primarily Urban	Most U.S. locations	Primarily urban (but available everywhere based on geolocation)	Primarily urban (but available everywhere based on geolocation)

Protocol	Parties mutual swipe exposes the chat option	Parties mutual swipe exposes the chat option	Users do not swipe but are presented with a grid of potentials 3 wide and length is dependent on subscription level. They can chat with these users immediately	Parties mutual swipe exposes the chat option only women can initiate the conversation

Primary user group	Heterosexual	Heterosexual	Homosexual Men	Heterosexual

All users	Heterosexual, gay, lesbian	Heterosexual, gay, lesbian	Homosexual Men, Women (∼6%)	Heterosexual, gay, lesbian

Positioning	Dating	Dating	Sexual partners and Dating	Dating

Choice limitation	10–21 selections per day (exact number determined by an algorithm)	100 selections confirming interest per day for the free version	100 people to chat with for the free version	50–100 swipes, number could fluctuate based on user patterns of behavior

Additional limitation	If communication does not occur after a fixed period of time the match will expire	None	None	Imposed time limit on response Women have 24 h to initiate the conversation or the match expires. After women initiate, their matches have 24 h to respond or the match expires

User data source	Populated by Facebook. Users can fill out additional dedicate fields such as height, relationship preference, religious affiliation, etc.	Options: (1) populated by Facebook, (2) associated to phone number/email address and populated manually	Options: (1) populated by Facebook (2) associated to phone number/email address and populated manually	Options: (1) populated by Facebook (2) associated to phone number/email address and populated manually

User data fields (all of these attributes correspond to the data at the time of the study; The user interface may change)	Users can fill out additional dedicate fields such as height, relationship preference, religious affiliation, etc.	Users have an About me section they can fill with any information (not verified)	In addition to a basic about me, users can provide Height, weight, Tribe (this is what type a person is within the homosexual community), Body type, Ethnicity, Looking for, and Relationship status	Users have an About me section they can fill with any information. Now: There are additional fields for Height, smoking, desire for children, etc.

### Future Work

Additional work might focus on features that are not labeled in the data but could potentially be inferred (either from the imagery, natural language processing, or social network data) and enhance our understanding of a user’s early priorities. Visual cues that could be analyzed may involve the style of the images available on the dating app, whether a person smiles or not, and aesthetic matches between pairs.

Natural Language Processing (NLP) cues could refer to the style of language used in the communication, such as length, usage of graphical icons, typos, grammar, etc. Primarily, since our results show broader choice strategies across men/women in the early stage followed by a narrowing of choices to arrive at a specific EMR, we can suspect that numerous additional pieces of information are brought up during the conversation that navigate the convergence. It would be interesting to investigate the conversations with the goal of unpacking the cues that users send to each other to signal their preferences. This is especially interesting if the information is not overtly discussed (i.e., if no user specifically asks the other “what religion do you subscribe to?” but then ends up matching with people who share their religious beliefs). More complex learning that can potentially be inferred (i.e., whether the two individuals smoke, share an interest in similar music, vote similarly, or share earning capacity) by analyzing the profile information may be used to enhance our understanding of whether similar traits lead to higher effective matching.

More complex NLP analyses might be used to reveal differences between the stated preferences and true intentions for a partner (i.e., an individual says that they are interested in dating for the purpose of a long-term relationship but seem to behave as if they are interested in a casual encounter). Accordingly, we can see if the similarity in actual preferences yields higher matches than the similarity in stated preferences.

Furthermore, our analyses assumed that the exchange of information on the mobile dating app is likely to be an indication of an interest in a romantic relationship and the exchange of information was an explicit way to arrange a date. This is not certain. Therefore, a future direction could look at what proportion of a conversation that culminated in the exchange of communication information indeed reflected a desire to meet in person for a romantic purpose.

Social network data could be used to learn whether group influence (shared friends, shared experiences, status within a group, etc.) are predictors of a successful match between two individuals. This could even be used to learn whether a person’s friends are valuable predictors of a successful match when the individual does not select his/her partner themselves, as suggested in prior works (Gilbert et al., 2009).

Given that our results were compared primarily to other matching protocols such as speed dating, matchmaking or arranged marriages [but also to the recent work by Bruch and Newman (2018) which highly aligns with our demographics as it focused on online dating in urban cities within similar population and age groups], it is worthwhile to continue observing the trends and data from additional popular apps, especially ones that focus on demographics outside of the ones observed in our work (i.e., homosexual communities, rural communities, or communities outside of Western societies).

### Connection to Neural Models of Choice

Drift diffusion models are frequently used as a proxy for the way decision making is conducted by neural mechanisms in our brain. An illustration of such a decision making model depicts the aggregation of information about the choice from the moment a user’s eyes land on a user profile to the moment their fingers swipe the phone to reflect their choice. Detailing the steps involved: a user’s visual cortex receives incoming input from the eyes about the option they are faced with on the phone’s screen and processes the information in order to decide whether to swipe left or right. Neural sites in the brain aggregate the information from the eyes alongside other cognitive internal processes and utilize a directed random walk to navigate the relative decision value assessment (Krajbich et al., 2014). Accumulated information is internally evaluated and drives the process, according to the weights and values of certain attributes and, ultimately, approaches a threshold for a decision. Once the threshold is crossed the conscious choice is manifested.

**FIGURE 6:**
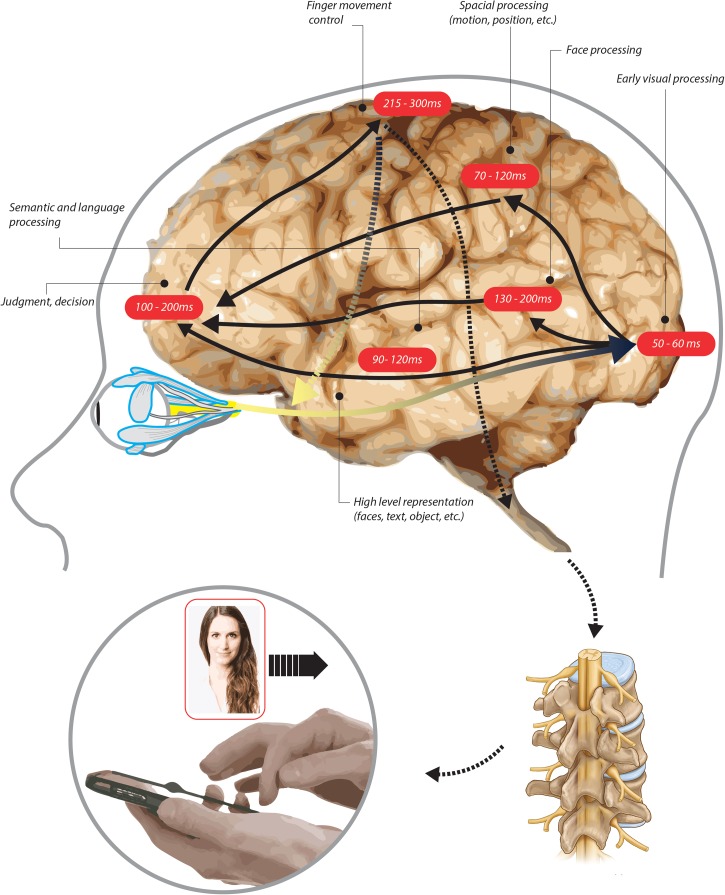
Illustration of the decision process and its latencies. Neuroscience estimates on the latency of information processing in the brain suggest that information from the retina is processed at the visual cortex within about 50 ms from the moment a person views an image (e.g., a picture of a person on the Hinge dating app). The information is then assessed in the “where” and “what” pathways within 50 ms more and, given that the images include the face of a person, is processed at the Fusiform Face Area within about 130 ms. Information from all pathways is ultimately aggregated at the prefrontal cortex where a judgment is potentially made. The choice is manifested as a motor control signal at the supplementary motor area which is communicated via the spinal cord to the fingers that swipe left/right to execute the selection. This process should typically last at least a third of a second. However, alternatives to the immediate choice could involve feedback from the Frontal Eye Field to the Superior Colliculus that drives an additional saccade of the eye to a different location on the screen (which, in turn, initiates a repetition of the processing), scrolling the phone screen for more information (using the motor controls) or additional high-level semantic processing (i.e., reading the text on the screen). All of those actions add additional time to the processing. Our results — combining the estimates of the decision making times using the DDM with an investigation of the average decision time allocated to all choices on the app — suggest that the initial selection happens rapidly and is based mostly on salient information. Given that the ultimate EMR reveals a strong preference for partners sharing attributes, information about the homophily could be gathered subconsciously from cues in the app that are indicative of preferences, or through the chat that happens throughout the communication.

An interesting venue to follow would be to apply the DDM in the context of neuroscience in similar ways to which other binary choices were previously tested in humans and apes (Gold and Shadlen, 2007) to see if mating preferences follow similar choice processes. This would allow us to track the decision making pathway with increased precision. For example, given prior estimates on the time it takes a visual cortex to process the input from the retina (approximately 120 ms; Mackay et al., 2012; see Figure 6), the additional time it takes the Fusiform Face Area (FFA) to process the face imagery (additional 40 ms), the time it takes the supplementary motor areas to plan, initiate, and execute the movement of the finger (additional 120 ms), and the estimate of additional processes, such as perception, reading, eye movement (in the case of scrolling), etc., we can narrow the time an individual user spent on the choices. In the example we illustrated here, subtracting the likely 500 ms for the perception/movement from a woman’s selection of a highly qualified (ranked above 8) man (average of 3.19 s; Figure 3), we can look at the rapid choice and suggest that the time allocated to the actual choice was close to 2.69 s, on average. This allows us to estimate the mental processing given to each decision and to incorporate that into our model.

Accordingly, an extension of this work could go beyond the realms of dating and onto studies of preferences. One could look at online mobile apps that match users for purposes of work, collaboration, or other social interactions. We might learn that a choice of, for example, hiring an employee, takes equal time whether elaborate thinking goes into it or whether it is done in fractions of a second. This might suggest that the notion of homophily and tendency for presence of similar characteristics are at the heart of more of our decisions.

## Conclusion

Discovery of potential romantic partners is currently dominated by mobile apps. These apps rely on similar methods for choosing a partner and offer a set of properties by which one can select and identify a potential match. While the information offered about an individual might differ across platforms, the majority of dating apps focus on a combination of visual imagery and a small number of features describing a user’s background and intentions. Algorithms for improved matching and the promise to help a user find their ideal match make the online dating industry flourish and occupy hours of some users’ days.

Combining our assessment of the early decision with the ultimate EMR, our results suggest that the saying “opposites attract” might not be true. On the contrary, individuals seem to gravitate toward partners that share traits with them. This is consistent with research that tested similarities between individuals in the context of choices, dating (Fiore and Donath, 2005; Skopek et al., 2010; Anderson et al., 2014), voting (Graham et al., 2009; Barnett and Cerf, 2018), and behavior (McPherson et al., 2001). Some works have linked the preference toward like-minded individuals or people who share key attributes with others to evolution, and to genetics. Some studies even suggest that identical twins who were separated at birth end up sharing some personality traits, behaviors, and preferences years later (Plomin et al., 1994; Jang et al., 1996; Rhee and Waldman, 2002). However, looking at datasets of the size we show here was challenging up until recently.

Thanks to the popularity of mobile apps that increase the amount of labeled data, and owing to the fact that the era of big data offers a set of readily available tools for loading and analyzing large datasets, we could investigate preferences in the context of dating on an unprecedented scale.

Our work contributes to the knowledge on mating choices in multiple ways. First, we show which parameters contribute to a likely match and their weights. Second, we show that a choice to move forward with interacting with a person or rejecting them can be estimated using a simple binary decision making model. Third, we show that, while the experience of online dating is quite different than that of other types of dating (i.e., in person meeting, or speed dating), the outcomes are similar. That is, in the course of seconds of exposure to a potential date, users are able to make a choice that parallels the one they would have made if they met the person in, for example, a bar. This suggests that online dating apps offer an advantage compared to offline methods of dating in scale. Because the pool of compatible partners increases dramatically, one can increase the return on invested time and effort and focus on a pool of individuals that match their preferences from a broader set of options. Although people may spend little time interacting with each profile on online mobile apps, they actually learn a significant amount about each other. Given that the world we live in is heading toward a more fast-paced nature of media and consumption (Cutting et al., 2010), these dating apps might dominate the dating sphere in the future.

This study is the first to explore the phenomenon of effective matching and dating preferences at such a large scale. After reviewing more than 421 million potential matches and examining this collection of proprietary data, we were able not only to replicate and validate the results of previous works, but also further push our understanding to realms not explored previously.

As we continue to see more of the population moving toward the use of mobile dating, developers and algorithm designers who are interested in maximizing the effectiveness of potential matches should accordingly design around similarities. However, taken to the extreme, this can lend itself to exclusion by various demographic characteristics and increased convergence to echo-chambers. We would encounter partners with higher alignment but be less exposed to opposing views and diverse personality characteristics. In light of this risk, it may be beneficial for society if apps were to present both similar options as well as intentional diversity.

As our knowledge on the topic grows and we continue to explore the data that drives our relationships, it would be unsurprising if the next generation of digital supported dating technologies moved toward Machine Learning and Artificial Intelligence tools that would eliminate the need for us to make selections ourselves. These would, instead, learn our priorities, weigh our decision processes, and emulate them.

It has not escaped our notice that in such a future we may be able to discover an ideal partner with the same simplicity as other current online experiences (i.e., ordering food or purchasing products online). People would open an app, provide access to a collection of personal data, and moments later their ideal mate would appear looking to schedule a first meeting.

Accordingly, some researchers identify a change in dating and commitment altogether, which may lead to an entire shift in the structure and social construct of pairing (Finkel, 2017). As a society, we could use results such as the ones provided here to either redefine the meaning and expectations from a match or adjust our understanding of the purposes of a relationship to a reality where alternatives are always nearby and stability in relationships is less frequent.

## Author Contributions

DM conducted all the analyses. JL and MC wrote the manuscript and conducted some of the meta-analyses.

## Conflict of Interest Statement

DM is an employee of Hinge Inc. and has competing financial interest.

The remaining authors declare that the research was conducted in the absence of any commercial or financial relationships that could be construed as a potential conflict of interest.

## Footnotes

1 https://hinge.co/terms

2 The rationale behind using “four way” as a measure of interest is so that the criteria for effective matching will reflect an actual likely desire to interact. Given that the cost of swiping right on multiple profiles on mobile dating apps is low—allowing users to do so without the necessary need to effectively choose a partner—some users use strategies of selection that only start the evaluation process *after* both parties have expressed interest. Therefore, if one party realizes that they are not interested then they will not initiate a conversation. So, if both people initiated a conversation and responded to the other person’s communication, the mobile dating industry considers the communication effective.
