# Supplementary material for: Polar Similars: Using Massive Mobile Dating Data to Predict Synchronization and Similarity in Dating Preferences
Source: Front Psychol. 2019 Sep 6;10:2010. doi: 10.3389/fpsyg.2019.02010 (PMC6743509; doi:10.3389/fpsyg.2019.02010)
Supplement: Supplementary file 1 [file Data_Sheet_1.docx]

**POLAR SIMILARS: USING MASSIVE MOBILE DATING DATA TO PREDICT SYNCHRONIZATION AND SIMILARITY IN DATING PREFERENCES**

**SUPPLEMENTARY INFORMATION**

**Appendix 1: list of cities from which Hinge user data was collected**

Albany, NY

Atlanta, GA

Austin, TX

Boston, MA

Chicago, IL

Cincinnati, OH

Columbus, OH

Charlotte, NC

Cleveland, OH

Dallas, TX

Washington, DC

Denver, CO

Detroit, MI

Houston, TX

Indianapolis, IN

Kansas City, MO

Los Angeles, CA

London (UK)

Miami, FL

Minneapolis-St. Paul, MN

Mumbai (India)

New Orleans, LA

Nashville, TN

New York, NY

Omaha, NE

Orlando, FL

Philadelphia, PA

Phoenix, AZ

Pittsburgh, PA

Portland, OR

Raleigh, NC

San Diego, CA

Seattle, WA

San Francisco, CA

St. Louis, MO

Sydney (Australia)

Toronto (Canada)

Tampa, FL

**Appendix 2: list of colleges by divisions 1 NCAA sports conferences**

| School | Primary Conference |
| --- | --- |
| Binghamton University | America East Conference |
| Stony Brook University | America East Conference |
| University at Albany, SUNY | America East Conference |
| University of Hartford | America East Conference |
| University of Maine | America East Conference |
| University of Maryland, Baltimore County | America East Conference |
| University of Massachusetts Lowell | America East Conference |
| University of New Hampshire | America East Conference |
| University of Vermont | America East Conference |
| East Carolina University | American Athletic Conference |
| Southern Methodist University | American Athletic Conference |
| Temple University | American Athletic Conference |
| Tulane University | American Athletic Conference |
| University of Central Florida | American Athletic Conference |
| University of Cincinnati | American Athletic Conference |
| University of Connecticut | American Athletic Conference |
| University of Houston | American Athletic Conference |
| University of Memphis | American Athletic Conference |
| University of South Florida | American Athletic Conference |
| University of Tulsa | American Athletic Conference |
| Wichita State University | American Athletic Conference |
| Davidson College | Atlantic 10 Conference |
| Duquesne University | Atlantic 10 Conference |
| Fordham University | Atlantic 10 Conference |
| George Mason University | Atlantic 10 Conference |
| George Washington University | Atlantic 10 Conference |
| La Salle University | Atlantic 10 Conference |
| Saint Joseph's University | Atlantic 10 Conference |
| Saint Louis University | Atlantic 10 Conference |
| St Bonaventure University | Atlantic 10 Conference |
| University of Dayton | Atlantic 10 Conference |
| University of Massachusetts Amherst | Atlantic 10 Conference |
| University of Rhode Island | Atlantic 10 Conference |
| University of Richmond | Atlantic 10 Conference |
| Virginia Commonwealth University | Atlantic 10 Conference |
| Boston College | Atlantic Coast Conference |
| Clemson University | Atlantic Coast Conference |
| Duke University | Atlantic Coast Conference |
| Florida State University | Atlantic Coast Conference |
| Georgia Institute of Technology | Atlantic Coast Conference |
| North Carolina State University | Atlantic Coast Conference |
| Syracuse University | Atlantic Coast Conference |
| University of Louisville | Atlantic Coast Conference |
| University of Miami | Atlantic Coast Conference |
| University of North Carolina at Chapel Hill | Atlantic Coast Conference |
| University of Notre Dame | Atlantic Coast Conference |
| University of Pittsburgh | Atlantic Coast Conference |
| University of Virginia | Atlantic Coast Conference |
| Virginia Tech | Atlantic Coast Conference |
| Wake Forest University | Atlantic Coast Conference |
| Florida Gulf Coast University | Atlantic Sun Conference |
| Jacksonville University | Atlantic Sun Conference |
| Kennesaw State University | Atlantic Sun Conference |
| Lipscomb University | Atlantic Sun Conference |
| New Jersey Institute of Technology | Atlantic Sun Conference |
| Stetson University | Atlantic Sun Conference |
| University of North Florida | Atlantic Sun Conference |
| University of South Carolina Upstate | Atlantic Sun Conference |
| Baylor University | Big 12 Conference |
| Iowa State University | Big 12 Conference |
| Kansas State University | Big 12 Conference |
| Oklahoma State University–Stillwater | Big 12 Conference |
| Texas Christian University | Big 12 Conference |
| Texas Tech University | Big 12 Conference |
| University of Kansas | Big 12 Conference |
| University of Oklahoma | Big 12 Conference |
| University of Texas at Austin | Big 12 Conference |
| West Virginia University | Big 12 Conference |
| Butler University | Big East Conference |
| Creighton University | Big East Conference |
| DePaul University | Big East Conference |
| Georgetown University | Big East Conference |
| Marquette University | Big East Conference |
| Providence College | Big East Conference |
| Seton Hall University | Big East Conference |
| St John's University | Big East Conference |
| Villanova University | Big East Conference |
| Xavier University | Big East Conference |
| California State University, Sacramento | Big Sky Conference |
| Eastern Washington University | Big Sky Conference |
| Idaho State University | Big Sky Conference |
| Montana State University | Big Sky Conference |
| Northern Arizona University | Big Sky Conference |
| Portland State University | Big Sky Conference |
| Southern Utah University | Big Sky Conference |
| University of Idaho | Big Sky Conference |
| University of Montana | Big Sky Conference |
| University of North Dakota | Big Sky Conference |
| University of Northern Colorado | Big Sky Conference |
| Weber State University | Big Sky Conference |
| Campbell University | Big South Conference |
| Charleston Southern University | Big South Conference |
| Gardner–Webb University | Big South Conference |
| High Point University | Big South Conference |
| Liberty University | Big South Conference |
| Longwood University | Big South Conference |
| Presbyterian College | Big South Conference |
| Radford University | Big South Conference |
| University of North Carolina at Asheville | Big South Conference |
| Winthrop University | Big South Conference |
| Indiana University | Big Ten Conference |
| Michigan State University | Big Ten Conference |
| Northwestern University | Big Ten Conference |
| Pennsylvania State University | Big Ten Conference |
| Purdue University | Big Ten Conference |
| Rutgers University | Big Ten Conference |
| The Ohio State University | Big Ten Conference |
| University of Illinois at Urbana–Champaign | Big Ten Conference |
| University of Iowa | Big Ten Conference |
| University of Maryland, College Park | Big Ten Conference |
| University of Michigan | Big Ten Conference |
| University of Minnesota | Big Ten Conference |
| University of Nebraska–Lincoln | Big Ten Conference |
| University of Wisconsin–Madison | Big Ten Conference |
| California Polytechnic State University | Big West Conference |
| California State University, Fullerton | Big West Conference |
| California State University, Long Beach | Big West Conference |
| California State University, Northridge | Big West Conference |
| University of California, Davis | Big West Conference |
| University of California, Irvine | Big West Conference |
| University of California, Riverside | Big West Conference |
| University of California, Santa Barbara | Big West Conference |
| University of Hawaii at Manoa | Big West Conference |
| College of Charleston | Colonial Athletic Association |
| College of William & Mary | Colonial Athletic Association |
| Drexel University | Colonial Athletic Association |
| Elon University | Colonial Athletic Association |
| Hofstra University | Colonial Athletic Association |
| James Madison University | Colonial Athletic Association |
| Northeastern University | Colonial Athletic Association |
| Towson University | Colonial Athletic Association |
| University of Delaware | Colonial Athletic Association |
| University of North Carolina at Wilmington | Colonial Athletic Association |
| Florida Atlantic University | Conference USA |
| Florida International University | Conference USA |
| Louisiana Tech University | Conference USA |
| Marshall University | Conference USA |
| Middle Tennessee State University | Conference USA |
| Old Dominion University | Conference USA |
| Rice University | Conference USA |
| University of Alabama at Birmingham | Conference USA |
| University of North Carolina at Charlotte | Conference USA |
| University of North Texas | Conference USA |
| University of Southern Mississippi | Conference USA |
| University of Texas at El Paso | Conference USA |
| University of Texas at San Antonio | Conference USA |
| Western Kentucky University | Conference USA |
| Cleveland State University | Horizon League |
| Indiana University – Purdue University Indianapolis | Horizon League |
| Northern Kentucky University | Horizon League |
| Oakland University | Horizon League |
| University of Detroit Mercy | Horizon League |
| University of Illinois at Chicago | Horizon League |
| University of Wisconsin–Green Bay | Horizon League |
| University of Wisconsin–Milwaukee | Horizon League |
| Wright State University | Horizon League |
| Youngstown State University | Horizon League |
| Brown University | Ivy League |
| Columbia University | Ivy League |
| Cornell University | Ivy League |
| Dartmouth College | Ivy League |
| Harvard University | Ivy League |
| Princeton University | Ivy League |
| University of Pennsylvania | Ivy League |
| Yale University | Ivy League |
| Canisius College | Metro Atlantic Athletic Conference |
| Fairfield University | Metro Atlantic Athletic Conference |
| Iona College | Metro Atlantic Athletic Conference |
| Manhattan College | Metro Atlantic Athletic Conference |
| Marist College | Metro Atlantic Athletic Conference |
| Monmouth University | Metro Atlantic Athletic Conference |
| Niagara University | Metro Atlantic Athletic Conference |
| Quinnipiac University | Metro Atlantic Athletic Conference |
| Rider University | Metro Atlantic Athletic Conference |
| Saint Peter's University | Metro Atlantic Athletic Conference |
| Siena College | Metro Atlantic Athletic Conference |
| Ball State University | Mid-American Conference |
| Bowling Green State University | Mid-American Conference |
| Central Michigan University | Mid-American Conference |
| Eastern Michigan University | Mid-American Conference |
| Kent State University | Mid-American Conference |
| Miami University | Mid-American Conference |
| Northern Illinois University | Mid-American Conference |
| Ohio University | Mid-American Conference |
| University at Buffalo | Mid-American Conference |
| University of Akron | Mid-American Conference |
| University of Toledo | Mid-American Conference |
| Western Michigan University | Mid-American Conference |
| Bethune-Cookman University | Mid-Eastern Athletic Conference |
| Coppin State University | Mid-Eastern Athletic Conference |
| Delaware State University | Mid-Eastern Athletic Conference |
| Florida A&M University | Mid-Eastern Athletic Conference |
| Hampton University | Mid-Eastern Athletic Conference |
| Howard University | Mid-Eastern Athletic Conference |
| Morgan State University | Mid-Eastern Athletic Conference |
| Norfolk State University | Mid-Eastern Athletic Conference |
| North Carolina Agricultural and Technical State University | Mid-Eastern Athletic Conference |
| North Carolina Central University | Mid-Eastern Athletic Conference |
| Savannah State University | Mid-Eastern Athletic Conference |
| South Carolina State University | Mid-Eastern Athletic Conference |
| University of Maryland Eastern Shore | Mid-Eastern Athletic Conference |
| Bradley University | Missouri Valley Conference |
| Drake University | Missouri Valley Conference |
| Illinois State University | Missouri Valley Conference |
| Indiana State University | Missouri Valley Conference |
| Loyola University Chicago | Missouri Valley Conference |
| Missouri State University | Missouri Valley Conference |
| Southern Illinois University Carbondale | Missouri Valley Conference |
| University of Evansville | Missouri Valley Conference |
| University of Northern Iowa | Missouri Valley Conference |
| Valparaiso University | Missouri Valley Conference |
| Boise State University | Mountain West Conference |
| California State University, Fresno | Mountain West Conference |
| Colorado State University | Mountain West Conference |
| San Diego State University | Mountain West Conference |
| San Jose State University | Mountain West Conference |
| United States Air Force Academy | Mountain West Conference |
| University of Nevada, Las Vegas | Mountain West Conference |
| University of Nevada, Reno | Mountain West Conference |
| University of New Mexico | Mountain West Conference |
| University of Wyoming | Mountain West Conference |
| Utah State University | Mountain West Conference |
| Bryant University | Northeast Conference |
| Central Connecticut State University | Northeast Conference |
| Fairleigh Dickinson University | Northeast Conference |
| Long Island University–Brooklyn | Northeast Conference |
| Mount St Mary's University | Northeast Conference |
| Robert Morris University | Northeast Conference |
| Sacred Heart University | Northeast Conference |
| Saint Francis University | Northeast Conference |
| St Francis College | Northeast Conference |
| Wagner College | Northeast Conference |
| Austin Peay State University | Ohio Valley Conference |
| Belmont University | Ohio Valley Conference |
| Eastern Illinois University | Ohio Valley Conference |
| Eastern Kentucky University | Ohio Valley Conference |
| Jacksonville State University | Ohio Valley Conference |
| Morehead State University | Ohio Valley Conference |
| Murray State University | Ohio Valley Conference |
| Southeast Missouri State University | Ohio Valley Conference |
| Southern Illinois University Edwardsville | Ohio Valley Conference |
| Tennessee State University | Ohio Valley Conference |
| Tennessee Technological University | Ohio Valley Conference |
| University of Tennessee at Martin | Ohio Valley Conference |
| Arizona State University | Pac-12 Conference |
| Oregon State University | Pac-12 Conference |
| Stanford University | Pac-12 Conference |
| University of Arizona | Pac-12 Conference |
| University of California, Berkeley | Pac-12 Conference |
| University of California, Los Angeles | Pac-12 Conference |
| University of Colorado Boulder | Pac-12 Conference |
| University of Oregon | Pac-12 Conference |
| University of Southern California | Pac-12 Conference |
| University of Utah | Pac-12 Conference |
| University of Washington | Pac-12 Conference |
| Washington State University | Pac-12 Conference |
| American University | Patriot League |
| Boston University | Patriot League |
| Bucknell University | Patriot League |
| Colgate University | Patriot League |
| College of the Holy Cross | Patriot League |
| Lafayette College | Patriot League |
| Lehigh University | Patriot League |
| Loyola University Maryland | Patriot League |
| United States Military Academy | Patriot League |
| United States Naval Academy (Navy) | Patriot League |
| Auburn University | Southeastern Conference |
| Louisiana State University | Southeastern Conference |
| Mississippi State University | Southeastern Conference |
| Texas A&M University | Southeastern Conference |
| University of Alabama | Southeastern Conference |
| University of Arkansas | Southeastern Conference |
| University of Florida | Southeastern Conference |
| University of Georgia | Southeastern Conference |
| University of Kentucky | Southeastern Conference |
| University of Mississippi | Southeastern Conference |
| University of Missouri | Southeastern Conference |
| University of South Carolina | Southeastern Conference |
| University of Tennessee | Southeastern Conference |
| Vanderbilt University | Southeastern Conference |
| East Tennessee State University | Southern Conference |
| Furman University | Southern Conference |
| Mercer University | Southern Conference |
| Samford University | Southern Conference |
| The Citadel | Southern Conference |
| University of North Carolina at Greensboro | Southern Conference |
| University of Tennessee at Chattanooga | Southern Conference |
| Virginia Military Institute | Southern Conference |
| Western Carolina University | Southern Conference |
| Wofford College | Southern Conference |
| Abilene Christian University | Southland Conference |
| Houston Baptist University | Southland Conference |
| Lamar University | Southland Conference |
| McNeese State University | Southland Conference |
| Nicholls State University | Southland Conference |
| Northwestern State University | Southland Conference |
| Sam Houston State University | Southland Conference |
| Southeastern Louisiana University | Southland Conference |
| Stephen F Austin State University | Southland Conference |
| Texas A&M University–Corpus Christi | Southland Conference |
| University of Central Arkansas | Southland Conference |
| University of New Orleans | Southland Conference |
| University of the Incarnate Word | Southland Conference |
| Alabama Agricultural and Mechanical University | Southwestern Athletic Conference |
| Alabama State University | Southwestern Athletic Conference |
| Alcorn State University | Southwestern Athletic Conference |
| Grambling State University | Southwestern Athletic Conference |
| Jackson State University | Southwestern Athletic Conference |
| Mississippi Valley State University | Southwestern Athletic Conference |
| Prairie View A&M University | Southwestern Athletic Conference |
| Southern University | Southwestern Athletic Conference |
| Texas Southern University | Southwestern Athletic Conference |
| University of Arkansas at Pine Bluff | Southwestern Athletic Conference |
| Appalachian State University | Sun Belt Conference |
| Arkansas State University | Sun Belt Conference |
| Coastal Carolina University | Sun Belt Conference |
| Georgia Southern University | Sun Belt Conference |
| Georgia State University | Sun Belt Conference |
| Texas State University | Sun Belt Conference |
| Troy University | Sun Belt Conference |
| University of Arkansas at Little Rock | Sun Belt Conference |
| University of Louisiana at Lafayette | Sun Belt Conference |
| University of Louisiana at Monroe | Sun Belt Conference |
| University of South Alabama | Sun Belt Conference |
| University of Texas at Arlington | Sun Belt Conference |
| Indiana University – Purdue University Fort Wayne | The Summit League |
| North Dakota State University | The Summit League |
| Oral Roberts University | The Summit League |
| South Dakota State University | The Summit League |
| University of Denver | The Summit League |
| University of Nebraska Omaha | The Summit League |
| University of South Dakota | The Summit League |
| Western Illinois University | The Summit League |
| Brigham Young University | West Coast Conference |
| Gonzaga University | West Coast Conference |
| Loyola Marymount University | West Coast Conference |
| Pepperdine University | West Coast Conference |
| Saint Mary's College of California | West Coast Conference |
| Santa Clara University | West Coast Conference |
| University of Portland | West Coast Conference |
| University of San Diego | West Coast Conference |
| University of San Francisco | West Coast Conference |
| University of the Pacific | West Coast Conference |
| California State University, Bakersfield | Western Athletic Conference |
| Chicago State University | Western Athletic Conference |
| Grand Canyon University | Western Athletic Conference |
| New Mexico State University | Western Athletic Conference |
| Seattle University | Western Athletic Conference |
| University of Missouri–Kansas City | Western Athletic Conference |
| University of Texas Rio Grande Valley | Western Athletic Conference |
| Utah Valley University | Western Athletic Conference |

**Appendix 3: NCAA conferences not included in research due to low sample size**

Big Sky Conference

Big South Conference

Horizon League

America East Conference

The Summit League

Atlantic Sun Conference

Mid-Eastern Athletic Conference

Western Athletic Conference

Southwestern Athletic Conference
